# Antiphased dust deposition and productivity in the Antarctic Zone over 1.5 million years

**DOI:** 10.1038/s41467-022-29642-5

**Published:** 2022-04-19

**Authors:** Michael E. Weber, Ian Bailey, Sidney R. Hemming, Yasmina M. Martos, Brendan T. Reilly, Thomas A. Ronge, Stefanie Brachfeld, Trevor Williams, Maureen Raymo, Simon T. Belt, Lukas Smik, Hendrik Vogel, Victoria L. Peck, Linda Armbrecht, Alix Cage, Fabricio G. Cardillo, Zhiheng Du, Gerson Fauth, Christopher J. Fogwill, Marga Garcia, Marlo Garnsworthy, Anna Glüder, Michelle Guitard, Marcus Gutjahr, Iván Hernández-Almeida, Frida S. Hoem, Ji-Hwan Hwang, Mutsumi Iizuka, Yuji Kato, Bridget Kenlee, Suzanne OConnell, Lara F. Pérez, Osamu Seki, Lee Stevens, Lisa Tauxe, Shubham Tripathi, Jonathan Warnock, Xufeng Zheng

**Affiliations:** 1 Institute for Geosciences, Department of Geochemistry and Petrology, University of Bonn, Bonn, 53115 Germany; 2 Camborne School of Mines and Environmental Sustainability Institute, University of Exeter, Penryn Campus, Treliever Road, Cornwall, TR10 9FE UK; 3 Lamont-Doherty Earth Observatory, Columbia University, Palisades, NY 10964 USA; 4 NASA Goddard Space Flight Center, Planetary Magnetospheres Laboratory, Greenbelt, MD 20771 USA; 5 University of Maryland, Department of Astronomy, College Park, MD 20742 USA; 6 Scripps Institution of Oceanography, University of California San Diego, La Jolla, CA 92093 USA; 7 Alfred Wegener Institute, Helmholtz Center for Polar and Marine Research, Bremerhaven, 27568 Germany; 8 Earth and Environmental Studies, Montclair State University, Montclair, NJ 07043 USA; 9 International Ocean Discovery Program, Texas AM University, College Station, TX 77845 USA; 10 School of Geography, Earth and Environmental Sciences, University of Plymouth, Plymouth, PL4 8AA UK; 11 Institute of Geological Sciences & Oeschger Centre for Climate Change Research, University of Bern, Bern, 3012 Switzerland; 12 British Antarctic Survey, Cambridge, CB3 0ET UK; 13 Institute of Marine and Antarctic Studies (IMAS), University of Tasmania, Hobart, TAS 7904 Australia; 14 School of Geography, Geology and the Environment, Keele University, Staffordshire, ST5 5BG UK; 15 Departamento Oceanografia, Servicio de Hidrografia Naval, Ministerio de Defensa, Buenos Aires, C1270ABV Argentina; 16 State Key Laboratory of Cryospheric Science, Northwest Institute of Eco-Environment and Resources, Lanzhou, 730000 China; 17 Geology Program, University of Vale do Rio dos Sinos, San Leopoldo, RS 93022-750 Brazil; 18 School of Water, Energy and Environment, Cranfield University, Bedfordshire, MK43 0AL UK; 19 Andalusian Institute of Earth Science (CSIC-UGR), Armilla, Granada 18100 Spain; 20 Cadiz Oceanographic Centre, IEO-CSIC, Cádiz, 11003 Spain; 21 Wordy Bird Studio, Wakefield, RI 02879 USA; 22 College of Earth, Ocean, and Atmospheric Sciences, Oregon State University, Corvallis, OR 97331 USA; 23 College of Marine Science, University of South Florida, St. Petersburg, FL 33701 USA; 24 GEOMAR Helmholtz Centre for Ocean Research Kiel, Kiel, 24148 Germany; 25 Department of Earth Sciences, ETH Zurich, Sonneggstrasse 5, Zurich, 8092 Switzerland; 26 Department of Earth Science, Marine Palynology and Paleoceanography, Utrecht University, Utrecht, 3584 CB The Netherlands; 27 Division of Earth Environmental System Sciences, Pukyong National University, Busan, 48513 Republic of Korea; 28 Knowledge Engineering, Tokyo City University, Tokyo, Setagaya-ku, 158-0087 Japan; 29 Faculty of Life and Environmental Sciences, University of Tsukuba, Tsukuba, Ibaraki 305-8572 Japan; 30 Department of Earth Sciences, University of California Riverside, Riverside, CA 92521 USA; 31 Department of Earth and Environmental Sciences, Wesleyan University, Middletown, CT 06459 USA; 32 Department of Marine Geology, Geological Survey of Denmark and Greenland, Aarhus, DK-8000 Denmark; 33 Institute of Low Temperature Science, Hokkaido University, Sapporo, Hokkaido 060-0819 Japan; 34 American Museum of Natural History, 200 Central Park West, New York, NY 10024 USA; 35 Marine Stable Isotope Lab, National Centre for Polar and Ocean Research, Ministry of Earth Sciences, Vasco Da Gama, 403804 India; 36 Department of Geography, Geology, Environment and Planning, Indiana University of Pennsylvania, Indiana, PA 15705 USA; 37 State Key Laboratory of Marine Resource Utilization in South China Sea, Hainan University, Haikou, Hainan 570228 China

**Keywords:** Cryospheric science, Palaeoceanography

## Abstract

The Southern Ocean paleoceanography provides key insights into how iron fertilization and oceanic productivity developed through Pleistocene ice-ages and their role in influencing the carbon cycle. We report a high-resolution record of dust deposition and ocean productivity for the Antarctic Zone, close to the main dust source, Patagonia. Our deep-ocean records cover the last 1.5 Ma, thus doubling that from Antarctic ice-cores. We find a 5 to 15-fold increase in dust deposition during glacials and a 2 to 5-fold increase in biogenic silica deposition, reflecting higher ocean productivity during interglacials. This antiphasing persisted throughout the last 25 glacial cycles. Dust deposition became more pronounced across the Mid-Pleistocene Transition (MPT) in the Southern Hemisphere, with an abrupt shift suggesting more severe glaciations since ~0.9 Ma. Productivity was intermediate pre-MPT, lowest during the MPT and highest since 0.4 Ma. Generally, glacials experienced extended sea-ice cover, reduced bottom-water export and Weddell Gyre dynamics, which helped lower atmospheric CO_2_ levels.

## Abstract (web summary)

A new high-resolution record for the Antarctic Zone shows persistent anti-phasing of high interglacial ocean productivity and high glacial dust deposition, suggesting a close inter-hemispheric coupling of cryosphere, ocean and atmosphere.

## Introduction

**Fig. 1:**
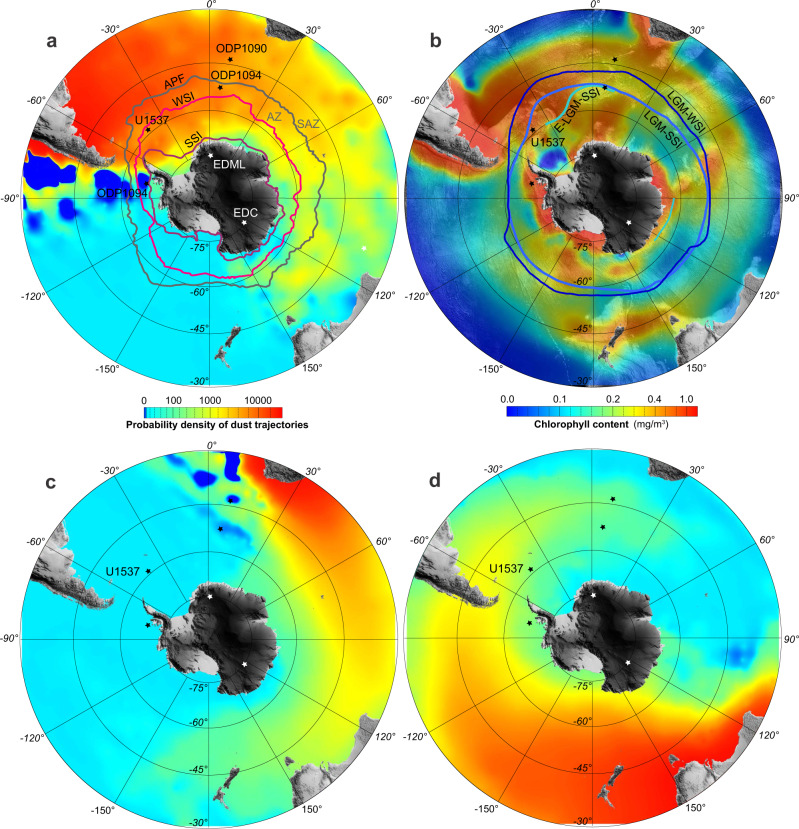
Antarctic dust provinces and productivity maps. Probability density map of dust trajectories from South America (**a**), South Africa (**c**), and Australia (**d**). SSI and WSI refer to austral summer (February) and winter (August) sea-ice extent, respectively, for 2019 (data from https://nsidc.org/data/seaice_index/archives). Antarctic Polar Front (APF) separates the Sub-Antarctic Zone (SAZ) in the north from the Antarctic Zone (AZ) in the south. **b** Average austral summer chlorophyll concentration from 2007 to 2015 (ref. ^18^) superimposed on bathymetry (model SRTM30_PLUS v7). Stars indicate sites referred to in this study. LGM-WSI, LGM-SSI and E-LGM-SSI refer to reconstructed last glacial maximum (LGM) winter sea-ice, CLIMAP summer sea-ice and EPILOG summer sea-ice extension, respectively (details see^61^).

A long-standing debate surrounding Earth’s climate system centers on whether ocean productivity enhanced by iron (Fe) fertilization from dust deposition^1^ fueled the biological carbon pump^2^, thus contributing to the glacial drawdown of up to 30% of atmospheric carbon dioxide (CO_2_) for storage in the deep ocean^3^. Although it is commonly accepted that dust deposition increased in both hemispheres during glacials, the fertilizing effect remains controversial and region specific^4^. For the sub-Antarctic zone (SAZ) of the Southern Ocean there is evidence for enhanced ocean productivity during glacials^5–7^, consistent with the Fe fertilization effect. In contrast, for the Antarctic zone (AZ)—the productive but Fe-limited Southern Ocean south of the Antarctic Polar Front (Fig. 1)—there is evidence for reduced glacial productivity^7,8^, although some studies suggest otherwise^9^, indicating that biosiliceous productivity within the AZ may be insensitive to increases in Fe supplied from dust, hence not contributing to glacial drawdown of atmospheric CO_2_. Accordingly, other processes such as increased sea-ice extent, reduced deep-water ventilation^10^, and enhanced water column stratification are likely important for the regulation of air-sea CO_2_ and heat exchange across the AZ during glacials^11,12^.

High upwelling rates are observed for the modern AZ, supplying nutrients and deeply sequestered CO_2_ to the surface ocean^4^. This could be the reason for enhanced interglacial productivity but due to Fe and light limitation, an inefficient biological pump might cause incomplete consumption of nutrients^13^. A more southerly position of Southern Hemisphere Westerly winds (SHW) over the core of the Antarctic Circumpolar Current (ACC) during interglacials, would support such enhanced upwelling^14^, and consequently lead to enhanced escape of CO_2_ to the atmosphere. Lower glacial productivity within the AZ could hence have been associated with more efficient nutrient consumption according to ^15^N measurements^15^ and a more northerly position of the SHW.

**Fig. 2:**
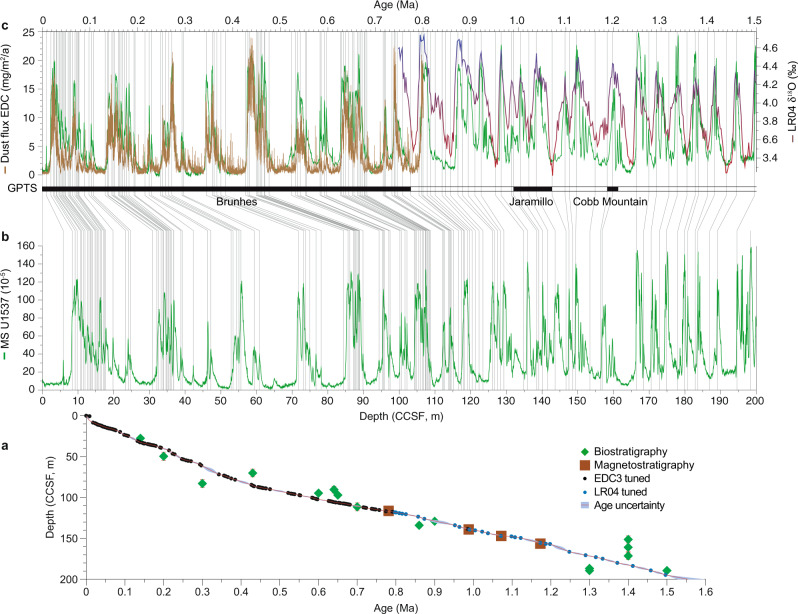
Age model of Site U1537. **a** Low-resolution age control for Site U1537 (Supplementary Table 1) is provided by magnetostratigraphy (5 reversals; brown squares; see also^76^) and biostratigraphy (18 datums; green diamonds with red bars indicating age uncertainties). Black and blue dots show the 133 tie points used to construct the high-resolution age model (Supplementary Table 2). Age uncertainties (blue shaded areas) were calculated using Undatable Matlab tools^77^ (“Methods”). Note that magnetic reversal boundaries were used as direct tie points, whereas the microfossil datums only provided limits. Note further that the sedimentation rates are generally higher since ~0.4 Ma (~20 cm/kyr) than before that time (~10 cm/kyr). **b** U1537 magnetic susceptibility (MS; green) record versus depth. **c** U1537 MS record versus age, dust flux record of ice core EDC based on the AICC2012 age scale^26^ (brown) and LR04 δ^18^O stack^23^ (red-blue). Underlain gray lines are the tie points for tuning maxima in MS to EDC dust flux peaks (0–0.8 Ma; black dots in age model) and to minima in the LR04 stack (0.8–1.5 Ma; blue dots in age model). Black-white pattern shows paleomagnetic reversals on the Geomagnetic Polarity Time Scale (GPTS). For 0–0.8 Ma ages are displayed on the AICC 2012 age scale^26^.

**Fig. 3:**
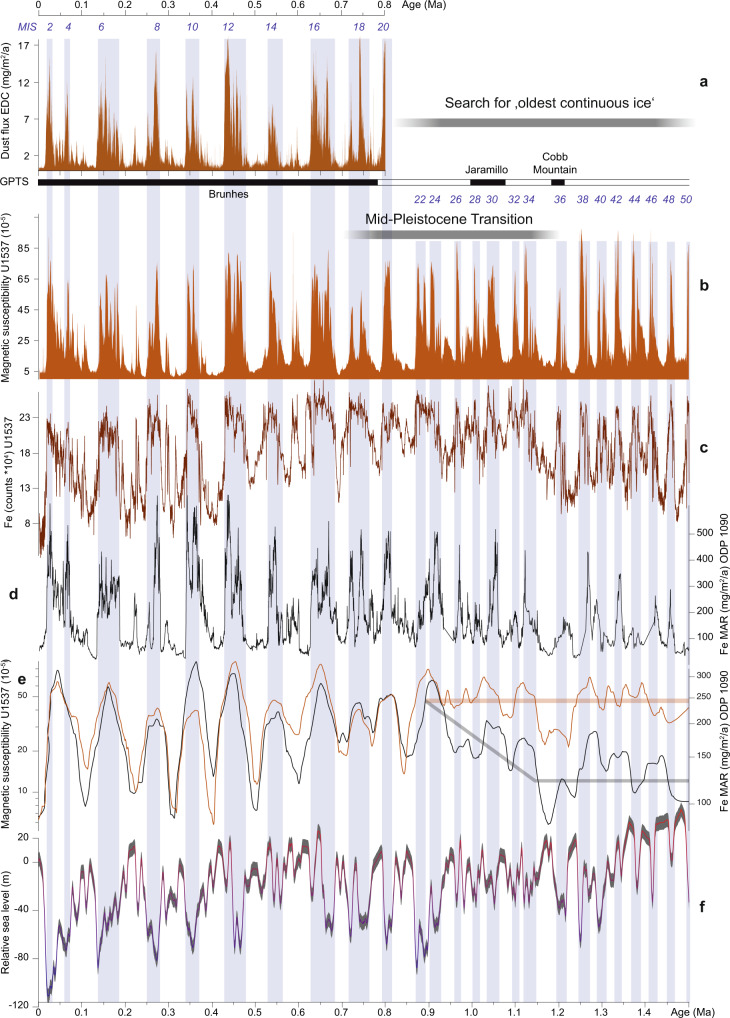
Antarctic dust deposition and global sea level over the past 1.5 Ma. **a** Dust flux record of the EPICA Dome C (EDC) ice core^19^. **b** Magnetic susceptibility (MS) record of Site U1537 with the Geomagnetic Polarity Time Scale (GPTS) above. **c** Fe counts of Site U1537. **d** Fe mass accumulation rates (MAR) of ODP Site 1090 from the SAZ in the South Atlantic^6^. **e** logarithmic plots of **b** and **d** using a 50-kyr smoothing. **f** Relative sea level (RSL) estimates from the Mediterranean Sea with probabilistic uncertainty estimates^49^. Underlain blue vertical bars indicate highs in MS during glacial Marine Isotopic Stages (MIS). Note the similarly enhanced values of Antarctic dust, MS and Fe during periods of low RSL. Note further the differences between ODP Site 1090 and U1537 until half way through the Mid-Pleistocene Transition (MPT).

**Fig. 4:**
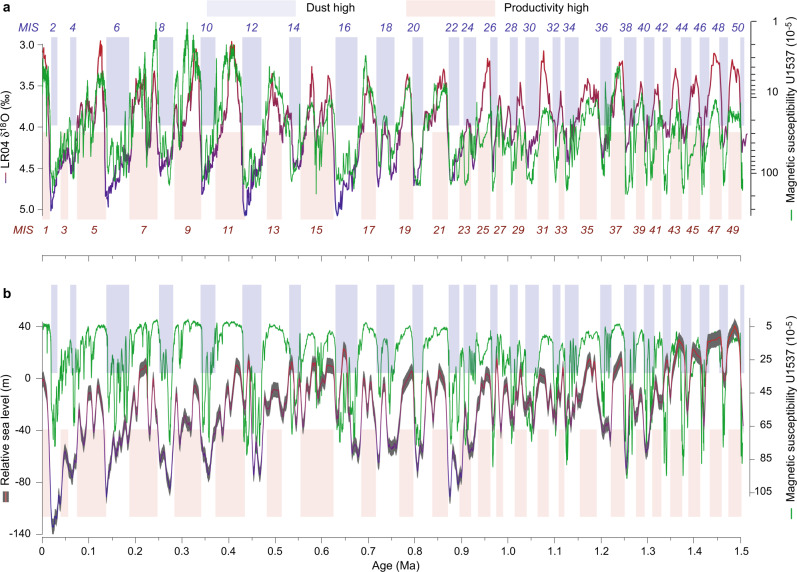
Climate coupling between Antarctic dust and Northern Hemisphere ice volume. Relationship between the dust proxy magnetic susceptibility (MS) record of Site U1537 and the global ice volume stack LR04 (ref. ^23^) (**a**) and relative sea level record from the Mediterranean^49^ (**b**). MIS refers to Marine Isotopic Stages. Note that dust deposition in the AZ, if plotted on a logarithmic scale, shows a very close relationship to variations in global ice volume. MS is similarly inversely correlated with relative sea level. Global ice volume and sea level are mainly driven by size variations in Northern Hemisphere ice sheets, pointing to a close interhemispheric coupling of cryospheric, oceanic and atmospheric processes.

**Fig. 5:**
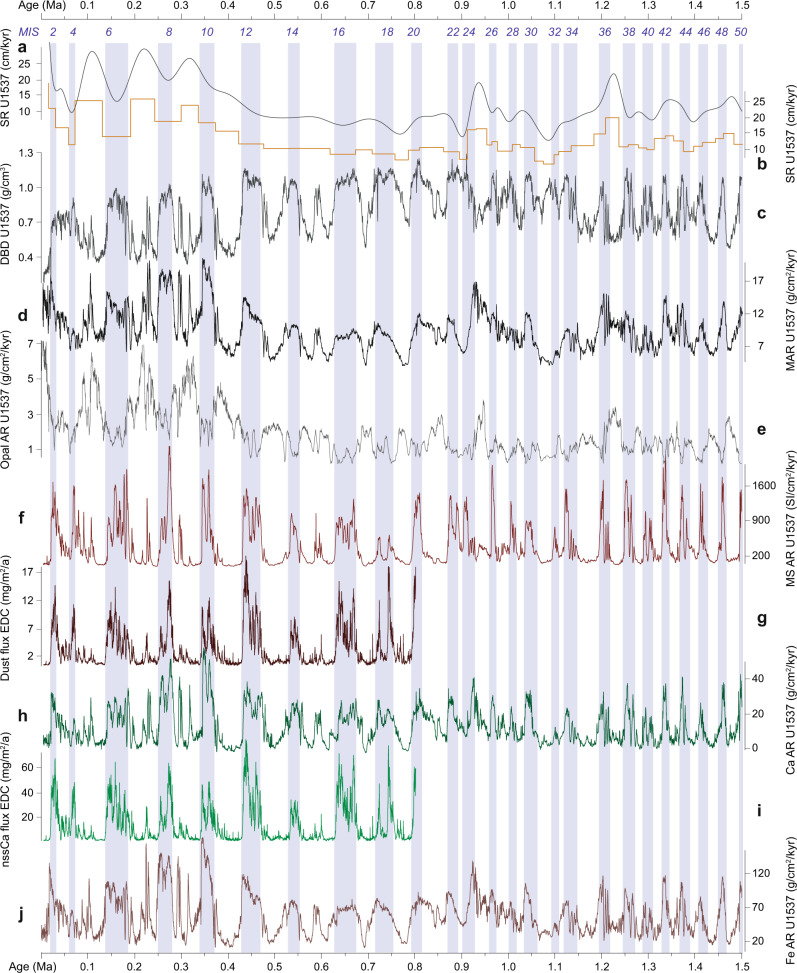
Accumulation rate trends of U1537 over the past 1.5 Ma. **a** Sedimentation rate (SR) calculated with a spline function for MIS boundaries. **b** SR variations displayed linearly between MIS boundaries. **c** Dry bulk density (DBD) calculated from moisture and density data^16^ and using an iterative procedure^72^. **d** Mass accumulation rate (MAR) calculated as the product of spline function SR and DBD. **e** Opal accumulation rate (AR) calculated as the product of MAR and the estimated % opal. **f** Magnetic susceptibility (MS) relative MAR in comparison to the EDC dust flux (**g**). **h** Ca MAR in comparison to nssCa flux of EDC (**i**). **j** Fe MAR. Underlain blue vertical bars indicate highs in MS during glacial Marine Isotopic Stages (MIS). EDC data from^26^. For 0–0.8 Ma ages are displayed on the AICC 2012 age scale^26^. Remaining legend as in Fig. 3.

**Fig. 6:**
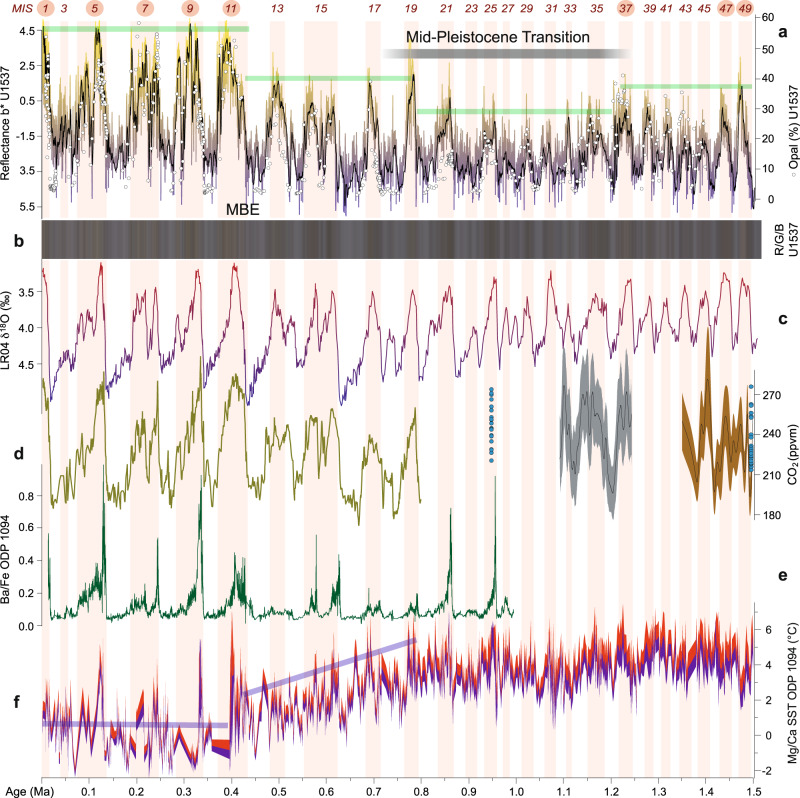
Paleoproductivity and atmospheric CO_2_ variation over the past 1.5 Ma. **a** Paleoproductivity proxy b* showing relative changes from terrigenous clay-rich sediment (blue) to opal-rich sediment (yellow). Right-hand scale gives opal content measured from Fourier Transformed Infrared Spectroscopy (FTIRS) (white dots) and calculated from b* (“Methods”). Underlain blue-yellow curve shows raw data; black curve gives 15-point smoothed average. **b** Color visualization of R/G/B data acquired with a line-scan camera (Methods). **c** Deep-sea isotopic stack LR04 for the past 1.5 Ma^23^. **d** Atmospheric CO_2_ records derived from a composite ice core record (green line), the Antarctic Allan Hills (blue dots) and δ^11^B-based estimates^52^ (gray and brown lines; see^51^ and references therein). **e** Ba/Fe record of ODP Site 1094 (ref. ^7^). **f** Magnesium calcium (Mg/Ca) based sea-surface temperature (SST) record of *Neogloboquadrina pachyderma* sinistral from ODP Site 1094 (ref. ^66^). Note diverging developments between MIS 19 at ~0.8 Ma and MIS 11 at ~0.4 Ma to other records. MBE. Mid-Brunhes event. Underlain pink vertical bars indicate highs in biogenic opal during interglacial Marine Isotopic Stages (MIS). Numbers with red circles indicate superproductivity interglacials. Note that higher atmospheric CO_2_ concentration correlates systematically with higher derived opal contents and thus higher biological productivity. Green lines indicate step-changes in inferred long-term paleoproductivity trends (see text for further discussion).

**Fig. 7:**
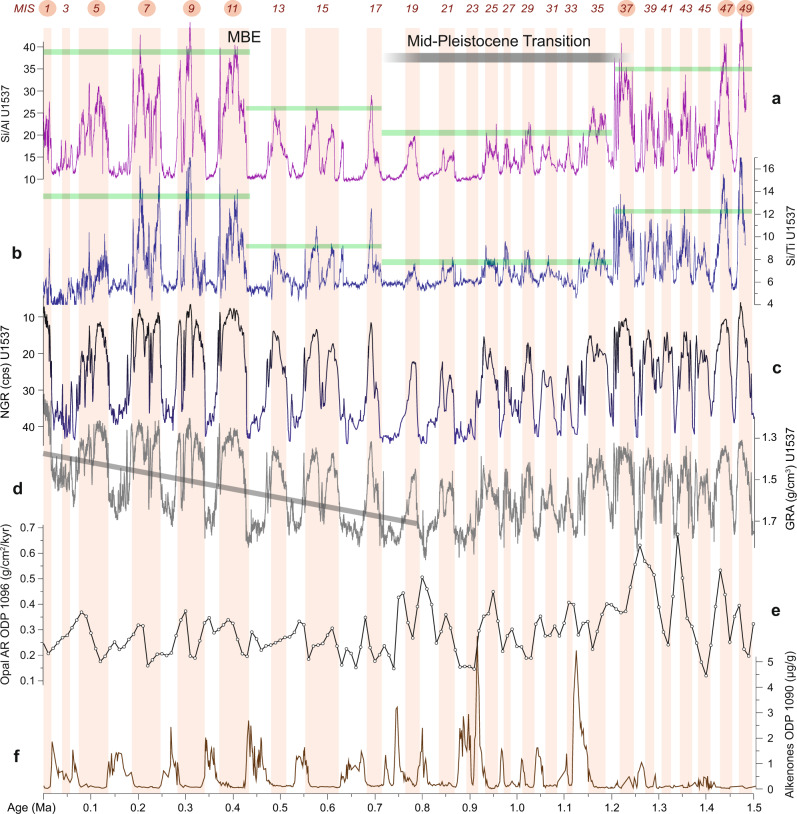
Paleoproductivity proxies and sedimentary facies over the past 1.5 Ma. **a** Si/Al ratio of Site U1537. **b** Si/Ti ratio. **c** Natural gamma radiation (NGR). **d** Gamma-ray density (GRA). Blue line shows trend of increasing GRA with depth due to de-watering and compaction in the upper ~120 m. Note that low GRA values are indicative of high opal content and high NGR values are typical for clay-rich deposits (Methods). **e** Opal MAR of ODP Site 1096 from the Pacific side of the Antarctic Peninsula^82^. **f** Alkenone content of ODP Site 1090 (ref. ^83^). Remaining legend as in Fig. 6. Note the general co-variance of each parameter on glacial-to-interglacial time scales. Note further that inferred paleoproductivity is consistently elevated during interglacials (pink vertical bars), especially during superproductivity periods (numbers with red circles).

International Ocean Discovery Program (IODP) Expedition 382 “Iceberg Alley”^16^ was aimed at improving our understanding of how the coupled Antarctic ice-ocean-atmosphere system evolved to the present state in the high latitude sea-ice region of the AZ (Fig. 1). At Scotia Sea Site U1537, we recovered the most continuous and highest resolution marine archive of dust and ocean productivity proxies obtained so far from near Antarctica for the Plio-Pleistocene (Figs. 2–7).

Dust deposition, as an Fe source, has been proposed as an important, large-scale driver in controlling natural climate variability for the Southern Ocean^17^, for which South America (Patagonia), Southern Africa and Australia are the major sources^18^ (Fig. 1). The longest record of Antarctic dust deposition comes from the 800,000 year-long EPICA Dome C (EDC) ice core^19^ (Fig. 1). The correlation of marine dust proxies to those from well-dated ice-cores represents a major step forward in the development of Southern Ocean chronologies for the Pacific^17^, the Atlantic^6^ and the Scotia Sea^20^, as dust deposition is near synchronous across much of the Southern Ocean and the Antarctic Ice Sheet. Hence, sedimentary records that pre-date continuous ice-core records such as ours will help to understand Pleistocene dust-climate couplings and how they contributed to regulate the carbon cycle.

## Results and discussions

### Chronology

A first, low-resolution, age model for Site U1537 is provided by shipboard magnetostratigraphy and biostratigraphy (Fig. 2; Methods, Supplementary Tables 1 and 2). Implications for rhythms of Antarctic climate since the Pliocene using this untuned age model are provided by^21^.

Earlier studies have identified a remarkable similarity in the structure of variability of magnetic susceptibility (MS) in cores from the Scotia Sea and the variability in dust records from Antarctic ice cores^22^. Independent age models from biostratigraphy (variations in *C. davisiana* abundance tied to LR04; ref. ^23^), radiocarbon dating and MS synchronization^24^, showed that age models from records in the Scotia Sea are “mutually consistent over their common ranges” on orbital and millennial time scales, and that fluctuations in marine MS and Antarctic dust concentration were synchronous. Studies on Sites MD07-3133 and MD07-3134 confirmed very close resemblance of the MS signal to the non-sea salt (nss) Ca^2+^ signal of the EDML ice core, a confident proxy for detrital dust in Antarctic ice cores^25^, and established the use of MS as high-resolution chronological control over the last glacial cycle^20^.

Hence, high-resolution age models developed here for Site U1537 follow the strategy developed for shorter cores^20^ by first synchronizing the marine dust proxy MS to the ice-core dust flux of the EDC ice core^19^ on the AICC 2012 age scale^26^ back to 0.8 Ma (black age tie points in Fig. 2). The synchronization shows a strong coupling between the two records, identifying dust-climate couplings as persistent glacial cycle feature. The relationship also reveals that there is a systematic association of dust maxima, natural gamma radiation (NGR), and gamma-ray densities (GRA) with glacial maxima, i.e. heaviest δ^18^O values in the LR04 record (Figs. 4 and 7), which is ground-truthed at magnetic reversals (Supplementary Fig. 4)^21^. We used this recognition in the next step and further tuned the lower part of the MS record (0.8–1.5 Ma), where no ice-core dust data exists, to glacials in the LR04 record (blue tie points in Fig. 2). The feasibility of this second step is supported by the notion that (1) glacial Fe maxima of Site U1537 align with glacial Fe maxima of independently dated Site ODP Site 1090 (ref. ^6^) (Fig. 3c, d) for that period along with maxima in NGR and GRA, (2) the age assignation is ground-truthed at paleomagnetic reversals^21^ (Supplementary Fig. 4) and (3) dust proxies show close covariation between ice volume and independently dated sea level changes when plotted logarithmically (Fig. 4) throughout the record, pointing to interhemispheric coupling.

### Dust proxies MS, Ca and Fe

The close resemblance between MS from Site U1537 and dust in the well-dated EDC ice core replicates earlier observations from short sediment cores in the study area^27,28^ that established such correlations for the last glacial cycle between MS and the EDML ice core. However, our records extend the preserved time to the last 25 glacial cycles. We similarly observe strong correlation between MS and Ca counts derived from XRF scanning (Methods; Supplementary Fig. 3) with dust deposition and non-sea salt Ca records from Antarctic ice-core records (Figs. 2 and 5). Since the Scotia Sea sites are located in deep water (≥3.2 km water depth) below the carbonate compensation depth, planktonic or benthic organisms producing carbonate shells are practically absent. Hence, Ca can be considered primarily of terrigenous origin. Fe weight % and flux rates have also been used to infer Antarctic dust-correlated atmospheric transport to the Pacific Ocean^17^ and the southeast Atlantic Ocean^6^. XRF-measured Fe counts from Site U1537 show a similarly close relationship to Antarctic dust patterns over the entire length of the ice-core record (i.e. 0.8 Ma; Figs. 3 and 5).

Overall, variations of terrigenous components MS, Ca and Fe in Site U1537 closely resemble all currently established dust tracers in the Southern Hemisphere with Antarctic dust maxima. All the components exhibit very spiky, high-amplitude patterns with low base levels during interglacials and 10–25 times higher values during glacials. We note that MS peaks are higher in the point sensor data (Supplementary Fig. 1) because of the higher spatial resolution of the measurement, implying that the glacial-to-interglacial amplitude might even be slightly higher. Even very fine details are reproduced between the marine and the ice-core records (e.g. Fig. 3a, b). These observations support the interpretation that the temporal variability of terrigenous components in marine sediments of the Scotia Sea are primarily controlled by the same large-scale atmospheric transport processes that deposit dust on Antarctica, and are a proxy for dust transport. Condensation of water, rainout and progressive polar amplification within the hydrologic cycle could provide such a processes accounting for the global spatial pattern of aerosol changes recorded in both marine sediment and ice^29^.

The proportion of terrigenous sediments in Southern Ocean cores that are of windblown origin is difficult to determine because there are few provenance studies and large uncertainties in the dust models, but previous studies (e.g.^17,30^) suggest that windblown dust may be an important fraction of the total. Thus, it is an open question if MS, Fe and Ca record windblown dust only, or whether they also record terrigenous sediments advected by ocean currents which would have to be controlled ultimately by wind strength, or some combination thereof. Here we make an estimate of windblown dust deposition rates in the Scotia Sea based on published models and focusing factors, and compare this to the terrigenous accumulation rate at Site U1537. Modeled dust flux estimates for the south Scotia Sea^31^ are ~5 g/m^2^/year during the LGM. This compares to terrigenous accumulation at U1537 of ~130 g/m^2^/year during the LGM and 80 g/m^2^/year in the MIS 6 glacial maximum, where terrigenous accumulation is calculated as total mass accumulation rate (MAR) minus opal MAR (see Fig. 5d, e). Sediment focusing in drifts concentrates planktic microfossils and dust, relative to their production/accumulation at the sea surface above the site (for more details on sediment focusing and calculation of MAR see further below). Core MD77-3134 from Dove Basin has a ^230^Th-derived glacial focusing factor of 11 to 29 (for interglacicals those values are 5–13) (ref. ^23^), i.e. 11–29 times more sediment was added to the core site via lateral transport than by vertical settling through the water column. If we assume that the focusing factor at MD77-3134 is 16 and focusing at U1537 is about half that at nearby MD77-3134 (because the accumulation rate at U1537 is about half), then modeled windblown dust accumulation would be about 8 × 5 g/m^2^/year = 40 g/m^2^/year, implying that dust can be a significant fraction of the glacial terrigenous MAR at Site U1537 (30–50% in this example estimate). We emphasize that there is a large degree of uncertainty in modeled dust estimates, for example the only nearby dust observations, at King George Island, are greater than the present day modeled value by an order of magnitude^31^, and the focusing factor can be extended only very approximately to Site U1537. In contrast to glacial conditions, interglacial dust supply is modeled to be ~0.1 to 1 g/m^2^/year (refs. ^31,32^). The Holocene focusing factor of MD07-3134 is 5–13 (ref. ^28^), and if, for this calculation, the factor at Site U1537 is half that, 4, then the dust deposition at Site U1537 would be 0.4 to 4 g/m^2^/year, which is ~1–10% of the Holocene interglacial terrigenous accumulation (~45 g/m^2^/year), a much lower, and possibly negligible, proportion than for dust deposition in glacials. This back-of-envelope exercise shows that, while there is a large uncertainty, windblown dust can be a significant proportion of the total glacial terrigenous fraction in the southern Scotia Sea.

Lower glacial and higher interglacial contributions of ocean currents to the terrigenous fraction is also indicated by Weddell Gyre dynamics. Besides the ACC, the Weddell Sea is an important source of Antarctic glaciomarine material that is presently transported to the Scotia Sea through the Orkney Passage^33^ via the northern limb of the Weddell Gyre. However, authigenic (seawater-derived) Nd and Pb isotope records from the Atlantic sector of the Southern Ocean suggest absence of Weddell Sea derived Antarctic Bottom Water (AABW) export to the north during the last two glacial maxima^10^. Also, current speeds were sluggish during the LGM in the northern Weddell Sea, indicative of a slow-down of the Weddell Gyre^34^. This conclusion is further supported by strong gyre-opposing currents forming contourite drifts in the southeastern Weddell Sea during the LGM^35^. Since the gyre is primarily wind driven and sea-ice cover was extensive in the Weddell Sea^34^, circulation was likely much slower or even stalled during glacials, largely prohibiting export of AABW to Site U1537 in the Scotia Sea. During deglaciation, bottom water transport resumed^10^, current speeds increased by an order of magnitude in the northern Weddell Sea during the Holocene^34^ and gyre-opposing bottom currents ceased in the southeastern Weddell Sea^35^, indicating together a declining sea-ice cover and more southerly winds^36^, and a generally stronger Weddell Gyre, capable of transporting a higher fraction of fine-grained, glaciomarine material to the Scotia Sea during interglacials. We should add that re-deposition of turbidity currents—often a major constituent of sediment drifts—is unlikely to have reached Dove Basin because of the South Orkney Trough between the continent and our sites.

Regardless whether terrigenous material is transported by the Weddell Gyre or the ACC, of all grain size fractions present in deep marine sediments, the clay fraction is most efficiently advected within ocean currents^37^. The contribution of these particles to the MS record depends strongly on their mineralogy. If composed of paramagnetic clay minerals, their contribution to the MS signal would be low. The provenance of terrigenous sediments in the Scotia Sea, using the detrital sedimentary Nd isotopic composition as a tracer, has a more Antarctic (less radiogenic) source during interglacials, and a more Patagonian and Antarctic Peninsula source during glacials^38,39^. Dust provenance studies from East Antarctic ice cores indicate Patagonia as the main dust supplier throughout the last 800 ka^40^. This suggests that windblown dust from Patagonia is a realistic terrigenous source during glacial climates for Dove Basin.

The median grain size by mass of desert eolian dust today is ~8 µm^41^, with sizes extending up to 20 µm, and only minor dust proportions in the <2 µm clay size fraction. Grain sizes >5 µm compose up to 50% of the total dust in the present day at Berkner Island of the Filchner-Rønne Ice Shelf in the southern Weddell Sea^42^. Trajectory studies identified two Patagonian dust sources with the southernmost area around San Julian’s Great Depression as the dominant dust source^43^. Dust transport times from Patagonia may be 1 day for our sites^31^ and up to 7 days for East Antarctic ice cores^43^. Since the Scotia Sea is located in the trajectory path of atmospheric transport from South America to East Antarctica, there is strong evidence that the eolian record of Site U1537 mostly originated from Patagonia.

A northward shift of the SHW during glacials has been suggested earlier^44^, implying that only then were the strongest SHW located over Patagonia. During glacial termination, the wind belt might have shifted south^14^, leaving Patagonia outside the center of high wind speeds during interglacials. However, the strength and position of the SHW is debated^45^. In any case, a strong glacial-to-interglacial gradient in dust transport is picked up by all dust proxy records at Site U1537 over each of the last 25 glacial terminations (Figs. 3–5).

Taken together, the evidence presented above suggests that windblown dust is the major contribution to records of MS, Ca and Fe, and is a significant mass contribution to glacial terrigenous sediments in Dove Basin. This is also supported by the fact that MS, Fe, and Ca mimic past Antarctic dust variability of the EDC ice core so closely (correlation coefficient r is 0.8 to 0.9), leaving only 10–20 % of the remaining variability to be driven by independent processes.

The close correlation of dust proxies MS, Fe and Ca with Antarctic glacial dust indicates that oceanic processes (Weddell Gyre and ACC dynamics) and atmospheric processes (SHW dynamics and the hydrologic cycle) are coupled on orbital and millennial scales. Interhemispheric ice-sheet ocean connections were coupled on orbital time scales mainly through sea-level forcing^46^, providing more glaciogenic material for both processes during glacials. On millennial time scales, strong atmospheric coupling is mainly observed for glacial maxima and early deglaciations^27^. During interglacials, this coupling weakens. Sediments from our study site have low dust content because rising sea levels during deglaciations flooded glacial outwash plains, diminished the area of dust availability, and increased the deposition area for terrigenous material on the continental shelves of Patagonia and Antarctica. Also, shifting or weakened SHW might have reduced the dust supply from Patagonia^14^. We should point out that Fe delivery by icebergs is unlikely a dominant fertilization source in the Scotia Sea because iceberg-rafted debris occurs primarily as pulses during glacial retreat after the LGM and is commonly lower during both glacials and interglacials in Iceberg Alley^20^.

### Long-term dust deposition including the Mid-Pleistocene Transition

**Fig. 8:**
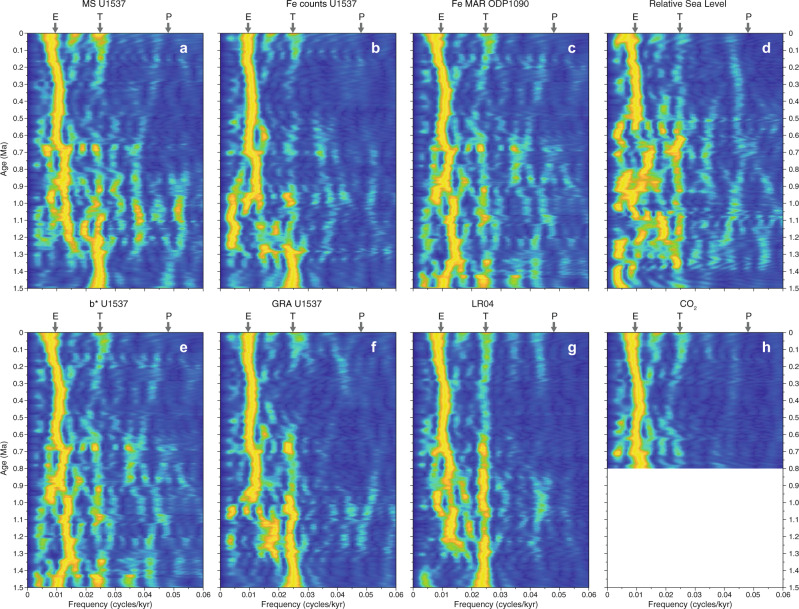
Evolutionary spectral analysis of dust and productivity time series for the past 1.5 Ma. **a** U1537 magnetic susceptibility (MS). **b** U1537 Fe counts of Site U1537. **c** ODP Site 1090 Fe mass accumulation rates (MAR)^6^. **d** Relative sea level of the Mediterranean^49^. **e** Color reflectance b* of U1537. **f** Gamma-ray density (GRA) of U1537. **g** Deep-sea isotopic stack LR04 (ref. ^23^). **h** Combined CO_2_ record from Antarctic ice cores^84^. Analysis was carried out with software ACYCLE^85^, using the fast Fourier transform, a window size of 300 kyr, and a 2.5 kyr moving window. E, T and P refer to the relative positions of eccentricity, tilt (obliquity) and precession, respectively. Note that MS and Fe counts of U1537 show a switch from 40-kyr to 100-kyr periodicity around 1.25 Ma at the beginning of the Mid-Pleistocene Transition (MPT). Additional important changes are documented half way through the MPT around 0.9 Ma and around 0.7 Ma at the end of the MPT (see text for details).

The record of Iceberg Alley dust proxy MS exhibits some peaks that are less pronounced or absent in the Antarctic ice core (Fig. 3a, b), e.g., between MIS 6–8, 8–10, 14–16, and 18–20. This is not surprising considering the close proximity of our sites to the Patagonian dust source (Fig. 1), where glacial outwash plains were widely exposed during periods of late glacial sea-level lowstands. In fact, the Patagonian land area almost doubled in size during the LGM^47^. Patagonia is the major dust source for the sites discussed here in the Atlantic sector of the Southern Ocean and Antarctic continent, i.e. IODP Site U1537, ODP Site 1090 and ice core EDC (Fig. 1a; see also^48^). The remaining dust sources in Southern Africa and Australia (data from^18^) are mainly relevant for the Indian and Pacific Ocean, respectively (Fig. 1c, d). Both marine and ice-core dust records exhibit the dominant 100,000-year (100-kyr) ice-age cyclicity of the Late Pleistocene (Fig. 8), implying common large-scale climate forcings, such as the hydrological cycle, latitudinal shifts or intensity variations of the SHW and regionally enhanced glaciogenic dust mobilization^17^.

The U1537 dust proxy records are strongly antiphased with an independently dated record of relative sea-level (RSL) change obtained in the Mediterranean^49^ throughout the last 1.5 Ma (MIS 2–50; Figs. 3 and 4). Generally, maxima in dust deposition are concentrated in glacial periods of lowered RSL. In detail, during MIS 2–6, 8, 10, and 20, low RSL and high dust occurred late in the glacial; during MIS 8 both occurred early. During MIS 12, however, dust shows an early and late glacial peak, whereas the RSL low is only pronounced early on. During MIS 14 there is a slight mismatch – a dust maximum occurred late in the glacial but the Mediterranean RSL was elevated at that time. MIS 16–18 both show double spikes in glacial sea-level lows and dust highs. Even smaller interglacial lowstands in RSL are accompanied by dust maxima, e.g. during MIS 15 and 17. Below ~MIS 30, the dust record contains many features that are no longer resolved in the lower-resolution RSL record of the Mediterranean, e.g. multiple spikes in MIS 38–46. The dust proxy records from Iceberg Alley also exhibit a remarkably close relationship to changes in global ice volume (Fig. 4), dominated by the Northern Hemisphere, at least since MIS 22.

Significantly, our marine dust proxy records span the Mid-Pleistocene Transition (MPT; ~1.25–0.7 million years (Ma) ago) at high resolution in the AZ. This is a critical period when the dominant glacial-interglacial cyclicity shifted from a 41-kyr to a 100-kyr periodicity (Fig. 8), despite no major changes in orbital forcing^50^. The MPT has hitherto not been recovered continuously in Antarctic ice cores; however, suitable locations have been recently found in the interior of East Antarctica to drill ice as old as 1.5 Ma. Across the MPT, global climate cooled (Figs. 3 and 5) and Northern Hemisphere ice sheets likely thickened. Glacial CO_2_ concentrations decreased overall by ~30 ppm, although there is no high-resolution or continuous record^51^ (Fig. 6d). Together, this evidence points to reorganization of the climate system’s internal feedbacks as a major driver^52^.

Enhanced dust input into the Southern Ocean may have been an important driver in Northern Hemisphere glaciation with stepwise increases at ~2.7 Ma, and 1.25 Ma^6^. Our dust proxy records for Site U1537 exhibit almost exclusively 41-kyr power before ~1.25 Ma (Fig. 8), with an abrupt change to a co-existence of 41-kyr and 100-kyr frequencies until ~0.7 Ma, and dominantly 100-kyr power, thereafter. However, our dust proxy records close to the Patagonian source show the same repetitive high-amplitude variability before, during, and after the MPT, with a 5 to 15-fold increase in concentration during glacials, depending on the dust proxy, and minima during interglacials (Fig. 3). This is different from the open SAZ, where the 100-kyr component is already present before the MPT (Fig. 8c) and dust deposition increased from ~1.15 Ma to ~0.9 Ma (MIS 34–22; see gray trend line in Fig. 3e), whereas in the AZ both glacial and interglacial dust levels were higher throughout the MPT. After ~0.9 Ma, both records vary more synchronously. This could be related to an abrupt increase in Antarctic ice volume at the onset of MIS 22 (ref. ^53^) providing a larger and more glaciated Patagonian dust source. It could also imply that glacial-to-interglacial latitudinal shifts or intensity variations in SHW or the hydrological cycle were more important in the western South Atlantic close to the dust source than amplitude changes in sea level and ice volume before and throughout the MPT, whereas in the SAZ dust deposition increased along with global ice volume during the MPT (Fig. 3e).

Glacial-to-interglacial sea-level amplitudes, as inferred from planktic δ^18^O values in the Mediterranean Sea^49^, also increased since ~0.9 Ma (Fig. 3f). We see the establishment of dominant 100-kyr cycles around MIS 22 for dust proxies MS and Fe at Site U1537 (Fig. 8a, b). Also, the coupling between global ice volume and dust is specifically strong since MIS 22 half way through the MPT (Fig. 4a), which is also true for the SAZ. Accordingly, similar patterns in dust deposition emerged across the Southern Hemisphere since ~0.9 Ma, and varied stronger on glacial-to-interglacial times in sync with increased ice-volume and sea-level changes. Such common changes provide compelling evidence for the interhemispheric coupling of cryospheric, atmospheric and oceanic processes.

### Paleoproductivity proxies

Increased export production in oceans is effective for CO_2_ removal from the atmosphere, provided sufficient light and nutrients are available^1^. Presently, Site U1537 is located in an area of relatively high chlorophyll-a concentration (Fig. 1b) and is in close proximity to the Patagonian dust source^18^. However, as established above, dust input into the Scotia Sea is substantially reduced today relative to glacial times. To gain deeper insight into dust-productivity couplings, we also measured biogenic opal—widely used as an indicator of enhanced upwelling and export ocean productivity^54^—and its high-resolution proxy, color reflectance yellow-blue component b* (Methods), at Site U1537 (Fig. 6).

Based on our glacial dust-climate synchronization, highs in estimated biogenic opal contents are systematically associated with interglacials at Site U1537 (Fig. 6a). The relationship is striking and holds through the entire record. While glacials are systematically dominated by terrigenous clays and silts and very low opal content (2–10%), interglacials are consistently dominated by massive diatomaceous clays to oozes 5–10 m thick (Methods), with opal content ~3–8 times higher (30–60%) than in glacial sediment.

Given that the overall sediment focusing is very pronounced at neighboring Site MD07-3134 (ref. ^28^) and that ^230^Th-normalization method works reliably only over ~the last two glacial cycles because of the ^230^Th half-life, conversion of the long U1537 dust and ocean productivity time series to flux data (Fig. 5) is flawed (Methods), although other constant flux proxies such as ^3^He (ref. ^55^) might shed light on sediment focusing in future studies. However, even if converted to fluxes, the dust proxies maintain their high-amplitude, glacial-to-interglacial variability and match the amplitude changes in ice cores, regardless (Fig. 5).

Normalization of XRF elemental scanning data can distinguish marine and terrigenous sources and is independent of sediment focusing. Si, for instance, is a primary constituent of biogenic opal but is also bound in terrigenous quartz. Al and Ti, however, commonly used as measures of terrigenous detrital input because their concentrations are largely unaffected by weathering and post-depositional alteration^56^. Hence, the ratio between Si and both of those elements should be indicative of paleoproductivity changes. Both proxies (Fig. 7a, b) show clearly enhanced productivity through all interglacials (MIS1–49), when diatomaceous oozes prevail. With slight deviations they follow the high-amplitude and long-term changes of b* and measured opal. Hence, XRF ratios too provide credible indication of interglacially enhanced paleoproductivity above our core site.

ODP Site 1096 from the Pacific side of the Antarctic Peninsula (Fig. 1) also shows higher opal fluxes during interglacials (Fig. 7e). Our interpretation of enhanced interglacial productivity is further supported by ship-board data indicating that the abundance of diatoms at Site U1537 were high, sea-ice related species were abundant, and preservation was good during interglacials relative to glacials^16^. Also, there is unlikely increased opal dissolution downcore (Methods). All in all, our data clearly indicate enhanced surface ocean productivity during interglacials (Figs. 6 and 7).

### Glacial to interglacial sea ice and CO_2_ variability

**Fig. 9:**
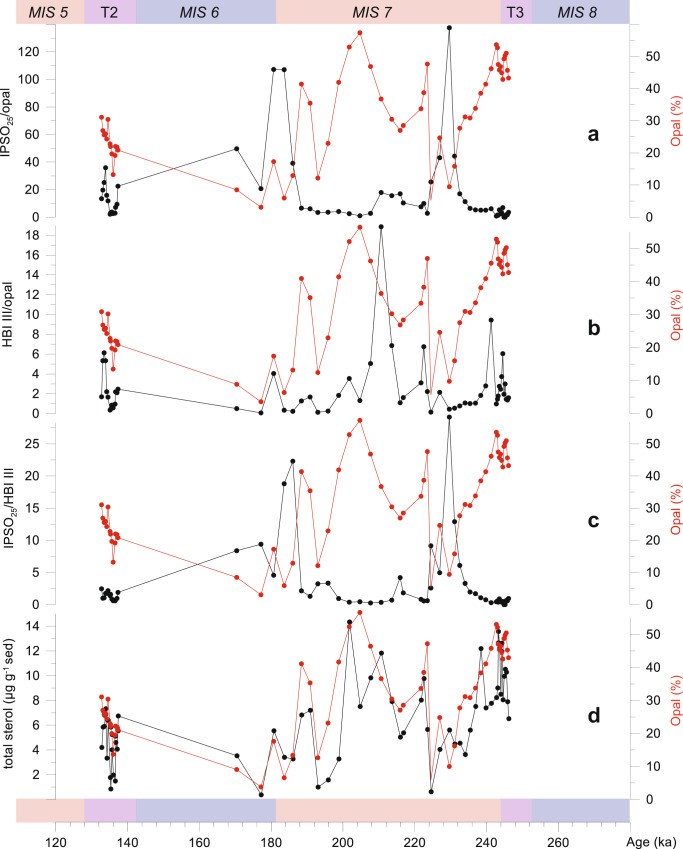
Paleoproductivity and sea-ice proxies for MIS 5–8. **a** Sea ice biomarker IPSO_25_/opal ratio^57^. **b** Biomarker HBI III/opal ratio. **c** IPSO_25_/HBI III ratio. **d** Total amount of sterols. Red curves show opal content. Measurements cover MIS 5–8 at Site U1537. T refers to glacial termination. Note that sea-ice indicators are inversely correlated to opal content and point to low sea-ice cover with open water conditions during times of elevated interglacial bioproductivity.

Evidence of seasonal glacial sea ice at Site U1537 is supported by the presence of the sea ice biomarker IPSO_25_ (ref. ^57^) almost through the entire core section studied (MIS 5–8; Fig. 9). However, when normalized to productivity data, i.e. biogenic opal—an approach recently adopted in the Ross Sea^58^ to resolve sea-ice dynamics over recent millennia—greater sea-ice coverage (high IPSO_25_) shows a clear out-of-phase relationship with higher biogenic opal productivity, demonstrating that sea-ice reduces productivity in this setting. In contrast, greater primary productivity is promoted by lower sea-ice coverage, as demonstrated by elevated opal contents and low IPSO_25_. The latter is further supported by analysis of a suite of phytosterols (individuals and totals; Fig. 9d), another major constituent of diatoms^59^, with the total sterol profile, in particular, closely following the opal data, as expected given the high % of diatoms. Similar outcomes are found when normalizing the IPSO_25_ concentrations to those of HBI III (Fig. 9c), a biomarker made by certain diatoms in the open waters of the marginal ice zone. Thus, preliminary biomarker-based sea-ice influence was greater during glacial times of low primary productivity and smaller during interglacial times of high productivity. An increase in interglacial export productivity has also been inferred for ODP Site 1094 (ref. ^7^). However, this site is located 2900 km farther east, in the open AZ of the Southern Ocean and north of the maximum winter sea-ice extent (Fig. 1b).

Site U1537 lies in an area that presently experiences some sea-ice cover during austral winter, but is ice-free for the remainder of the year (Fig. 1a). This strong seasonal gradient fuels biological productivity in the surface ocean, especially for seasonal sea-ice-adapted siliceous plankton^60^, and could hence explain higher interglacial ocean productivity. For the Last Glacial Maximum, the winter sea-ice boundary was displaced north of Site U1537, whereas the summer sea-ice boundary was likely near Site U1537 (ref. ^61^) (Fig. 1b). Under glacial conditions of more extensive sea-ice cover at Site U1537 (Fig. 9), atmosphere-ocean gas exchange would likely have been diminished^62^. Accordingly, enhanced glacial surface-water stratification in the AZ may have exerted a stronger control on atmospheric CO_2_ levels by limiting release of oceanic CO_2_ into the atmosphere compared with active drawdown of CO_2_ via export production in the SAZ^12^.

Such an increase in glacial sea-ice coverage would also have reduced deep-water ventilation in the spatially expanded AZ^63^ with only limited injection of nutrients from deep to surface waters, thereby reducing glacial productivity^4^. Further, the glacial-interglacial amplitude in atmospheric CO_2_ varies widely (up to 100 ppm) throughout the last 1.5 Ma (Fig. 6d) and the early Pleistocene before that. If the biological pump played an important role in glacial atmospheric CO_2_ drawdown in the Southern Ocean, the alkenone concentration of ODP Site 1090 should exhibit large glacial-interglacial fluctuations throughout; instead it is consistently low from 3.6 to 1.15 Ma, and only fluctuates thereafter (Fig. 7f). Furthermore, studies on the last glacial cycle conclude that observational evidence does not support the idea that large-scale changes in the marine biological pump was the dominant influence on atmospheric CO2 changes during glaciations^64^.

As such, biosiliceous productivity in the Scotia Sea appears insensitive to the increase in glacial Fe supply from dust, or may lack other micro-nutrients and was thus unlikely responsible for significant glacial drawdown in atmospheric CO_2_ (ref. ^8^) in the AZ. Instead, the net CO_2_ drawdown during glacials could have resulted from more efficient trapping of CO_2_ in the deep ocean by a combination of spatially expanded sea ice, longer mean deep water residence times in the deep sea, and enhanced surface-ocean stratification in the AZ, and active CO_2_ drawdown by increased productivity in the SAZ^7^. Our data support such a view.

### Long-term productivity history in Iceberg Alley

The glacial-interglacial pattern of inferred productivity changes generally follows global ice volume with an asymmetrical pacing of relatively fast changes from low to high ocean productivity at the begin of an interglacial, and a slow decline during warm periods when compared to the LR04 stack (e.g., MIS 1–9, 19, 21 and 25) (Figs. 6 and 7). However, some features are more symmetrical than in the isotopic stack, specifically in the older part of the opal record (e.g., MIS 11, 17, 35, 37, 47 and 49). This is why there is a general positive relation between b* (or derived opal contents) and LR04 but also some scattering (Supplementary Fig. 2). ODP Site 1094 from the open ocean AZ exhibits spikes in bioproductivity exclusively at the very beginning of interglacials, as inferred from Ba/Fe ratios (Fig. 6e). However, this site is located farther north, outside the winter sea-ice zone of the AZ (Fig. 1). It could therefore be that the more symmetrical highs in bioproductivity at Site U1537 are caused by the higher influence of the waning and waxing sea-ice coverage during interglacials.

Besides glacial-interglacial variability, the productivity record of Site U1537 shows four long-term, step changes as deduced from the opal, Si/Al and Si/Ti proxies (marked by green lines in Figs. 6a and 7a, b). Productivity is low and exhibits low-amplitude, high-frequency variability following the termination of the Olduvai magnetic subchron (at 1.778 Ma; Supplementary Fig. 1 and Supplementary Table 1). At the beginning of our reconstruction there are two distinct, high-amplitude cycles at MIS 49 and 47 (~1.487 Ma and ~1.427 Ma, respectively; Figs. 6 and 7). The next rather broad maximum centers around MIS 37 (~1.24 Ma), at the beginning of the MPT, when the amplitude of the 100-kyr component in the LR04 benthic isotopic stack increases, too^23^ (Figs. 6c and 8g), indicating that the boundary conditions driving glacial-interglacial transitions and sea-level^50^ changed. During the MPT, interglacial productivity was lowest at Site U1537 until MIS 19 (~0.77 Ma, Fig. 6a) or MIS 17 (~0.7 Ma; Fig. 7a, b), depending on the proxy, followed by a moderate increase at the end of the MPT. Although the inverse relationship of productivity and sea-ice coverage above Site U1537 may have played an important role in lowering atmospheric CO_2_ during glacials for both the 41-kyr and 100-kyr worlds, the long-term step changes exhibit four different plateaus and indicate that productivity and sea-ice changes were unlikely responsible for the 30 ppm additional CO_2_ drawdown during glacials across the MPT.

In this context, we highlight that MIS 31 (at ~1.07 Ma) is considered one of the so-called “superinterglacials” in the Northern Hemisphere and around Antarctica^65^, with elevated temperatures and potential instabilities for the Antarctic ice sheet. Although we do see elevated paleoproductivity during all interglacials, MIS 29–33 have very low peak values in the Scotia Sea. Our paleoproductivity records do, however, show the existence of superproductivity periods—those with distinctly elevated amplitudes, for MIS 1, 5–11, and tentatively for MIS 37 and 47–49 (see numbers with red circles in Figs. 6 and 7), indicative of very strong interglacial upwelling, especially since the Mid-Brunhes Event (MBE) at MIS 11.

The four interglacials following the MPT (MIS 19–13) all show increased opal amplitudes with plateau-like peaks (opal contents around 40%) until ~0.47 Ma. Sea-surface temperatures at Site ODP 1094 (ref. ^66^) became progressively colder between MIS 19 and 11 (~0.43 Ma) (see purple bars in Fig. 6f) during the period of “lukewarm” interglacials^7^, possible indicative of hemisphere-wide cooling.

After MIS 13 there is a final step-wise increase to the highest interglacial productivity values at Site U1537. The last five interglacials (MIS 11–1) all reach plateau-like highs and exhibit the largest glacial-to-interglacial amplitude in productivity changes. This is likely related to changes associated with the MBE, a marked global increase in climate variance during MIS 12 at ~0.43 Ma. Since MIS 12, ice-cores document interglacials with higher atmospheric CO_2_ levels (Fig. 6d), while δ^18^O records suggest that interglacials had higher sea levels and that the amplitude of the 100-kyr cycle was increased^67^. The last five interglacials have also shown increased Antarctic temperature and ice loss from the Wilkes Subglacial Basin^68^. In addition, increased interglacial ocean productivity has been inferred from opal and barium records in a number of sites from the continental margin of the Weddell Sea over the last 400 ka^69^. Interestingly, our paleoproductivity record from Site U1537 also shows a substantial increase in the amplitude of the 100-kyr cycle for these superproductivity periods since the MBE (Figs. 6–8) adjacent to the Antarctic Ice Sheet.

### Cryosphere-ocean-atmosphere couplings

Overall, we see a distinct increase in dust deposition during glacials but little indication for enhanced glacial CO_2_ drawdown by siliceous primary producers from the atmosphere through Fe fertilization in the high latitude AZ. Instead, we see clear evidence for spatially and temporally variable controls of ocean productivity and dust deposition across the Southern Ocean. Further, marine terrigenous deposition depended on the extent of bottom water export and Weddell Gyre dynamics.

In Iceberg Alley, ocean productivity increased during interglacials, especially during superproductivity periods before the MPT and after the MBE. We also identify a close relationship between dust proxies in Iceberg Alley and variations in global ice volume and sea level, both of which are driven mainly by ice-sheet size variations in the Northern Hemisphere. However, glacial dust deposition was high in the AZ throughout the last 1.5 Ma, while it increased in the SAZ during the MPT, indicative of increasingly stronger and farther reaching SHW or less regional differences in the hydrological cycle. Half way through the MPT, around 0.9 Ma, the coupling between global ice volume and dust became very strong and the glacial-to-interglacial sea-level amplitudes increased. Accordingly, we see a close interhemispheric coupling of cryospheric, oceanic and atmospheric processes in the AZ throughout the last 1.5 Ma.

The Scotia Sea records also highlight that paleoproductivity, SHW and sea-ice feedbacks play a crucial role in regulating atmospheric CO_2_, with northward movement of the sea-ice front and SHW during glacials reducing gas release from the ocean to the atmosphere across the AZ, and southward movement of the sea-ice front and SHW associated with higher upwelling and paleoproductivity as well as enhanced CO_2_ release to the atmosphere during interglacials, especially during superinterglacials. Also, reduction in glacial atmospheric CO_2_ but no additional CO_2_ drawdown through the MPT is consistent with our records.

## Methods

### Site location and depth construction

International Ocean Drilling Program (IODP) Site U1537 is located in the center of Iceberg Alley in the southern Scotia Sea (Fig. 1). It is 265 km northwest of the South Orkney Islands at 59° 6.65’ S, 40° 54.37’ W in 3713 m water depth. The site lies in the northeast part of Dove Basin on a ~1 km thick contourite drift^70^. Site MD07-3134 lies ~40 km southwest on the same contourite in 3663 m water depth and is located at 59° 25’ S, 41° 28’ W with a core length of 58.2 m (ref. ^27^). For this study we use the two longer holes that were drilled at Site U1537 with the Advanced Piston Corer. At Hole U1537A the upper 264.0 meters below seafloor (CSF-A) were drilled with a recovery of 268.9 m (102%). At Hole U1537D the upper 354.3 m CSF-A were drilled and recovered 349.0 m (99%). Both holes were retrieved during calm weather conditions and obtained virtually the same sedimentary record with major lithologic features occurring at similar depths in each hole (Supplementary Fig. 1).

Our scientific objectives require recovery of complete stratigraphic sections to the best extent possible to establish robust age controls (Fig. 2). Such a continuous sedimentary sequence cannot be recovered from a single hole because gaps exist between successive cores. We constructed a composite stratigraphic section, the splice, by combining stratigraphic sections from holes A and D (Supplementary Fig. 1) to produce a composite, and continuous record and depth scale (CCSF) (for guidelines see https://www.iodp.org/policies-and-guidelines/142-iodp-depth-scales-terminology-april-2011/file). Hence the start of our reconstruction at 1.5 Ma translates into ~183 m (CSF-A) in Hole U1537A, ~182 m (CSF-A) in Hole U1537D, and ~195 m (CCSF) for the splice of Site U1537 (ref. ^16^). All depths reported in this paper are in m CCSF.

### Non-destructive physical, optical and chemical measurements

The core sections were first analyzed onboard JOIDES Resolution with the Whole-Round Multisensor Logger at 2-cm intervals to determine MS (Figs. 3 and 4) with a loop sensor and gamma-ray density (GRA; Fig. 7). Then NGR (Fig. 7) was also determined on whole-round sections to collect spectral gamma-ray data at 2-cm intervals.

After core splitting, the Section Half Multisensor Logger (SHMSL) was used to measure MS again (Supplementary Fig. 1), this time with a Bartington surface scanning point sensor. Both loop and point sensors produced coherent and reliable results, with the point sensor showing more pronounced peaks owing to the smaller measurement footprint. For the dust reconstruction we used the whole-round sensor data in this study. Then color reflectance was also measured on the SHMSL using an Ocean Optics spectrophotometer at 2-cm resolution to obtain color information on the blue-yellow variability (b*; Fig. 6). Finally, a commercial line-scan camera lens (AF Micro Nikon; 60 mm; 1:2.8 D) was used to generate a line-scan image of each split half core, and R/G/B color values were retrieved from the image at 0.5–2-cm intervals, and plotted accordingly in Fig. 6b. For detailed descriptions of methodologies and procedures see^16,71,72^.

Postcruise, we also measured the distribution of chemical elements using an AVAATECH X-ray Fluorescence (XRF) Core Scanner along the splice of Site U1537 (measurement details see^73^). Results for each element are given as peak area intensities determined in total counts per second (cps). The measurement increment varied between 2 cm and 1 mm.

### Biogenic opal determination

Fourier Transform Infrared Spectroscopy (FTIRS) was used to quantify biogenic opal to obtain information on past changes in ocean productivity. We measured 616 samples covering the last 1.4 Ma (Fig. 6a), of which every 20th sample was measured twice in order to determine analytical precision/reproducibility for a sample batch. Prior to analysis 11 ± 0.05 mg powdered and dried sediment was homogenized along with 500 ± 0.05 mg spectroscopic grade and oven-dried (12 h at 200 °C) KBr (Uvasol©, Merck). Samples have undergone a dedicated treatment designed for porous and NaCl bearing marine sediments, which involves an additional drying step for 2 h at 200 °C of samples already placed in the measurement plate^74^. FTIR spectra were recorded by a Bruker Vertex 70, equipped with a MCT (mercury cadmium-telluride) detector, a KBr beam splitter, and a HTS-XT accessory unit. Each sample was scanned 64 times at a wavenumber resolution of 4 cm^−1^ (reciprocal centimeters) for the wavenumber range from 520 cm^−1^ to 3750 cm^−1^ with the aperture set to 8 mm in diffuse reflectance mode.

A partial least squares regression model was used based on synthetic sediment samples with known biogenic opal content^28^, which has been shown to produce reliable results with an accuracy as determined by means of the root mean square error of cross-validation of 4.7 % (in % bSi). The absolute percentage error (MAPE) would be ≤11% over the entire 0–100% opal range and ≤5.7% above 10% opal^74^, which is primarily an effect of scaling of the relative error of the calibration. Comparison of laboratory standards measured by means of the different wet chemical and the FTIRS method yielded very similar results^74^. Precision and reproducibility of the FTIRS method is high for the U1537A samples batch with a mean deviation of 3.4% and a range of 0–8.4% of the measured value.

FTIRS has been implemented successfully to reconstruct paleoproductivity on neighboring cores MD07-3133 and MD07-3134 for which FTIRS derived opal has been compared against opal measured by means of “conventional” leaching techniques^28^.

### Sea-ice biomarker measurements

Lipid analysis^75^ was carried with a slight modification to the extraction method. Briefly, freeze-dried samples (ca. 3–4 g) were saponified in a methanolic Potassium Hydroxide (KOH) solution (Methanol:MilliQ water (9:1,v/v); 5 % KOH) for 60 min (70 °C). Hexane (3 × 2 ml) was added to the cooled saponified content, with supernatant containing non-saponifiable lipids (NSLs) transferred to clean vials and dried over Nitrogen (N_2_, 25 °C). NSLs were then further fractionated using silica (60 200 µm, 0.5 g) column chromatography. HBIs were eluted with hexane (6 ml) and purified further using silver-ion chromatography (Discovery® Ag-Ion; ca. 0.1 g), with saturated compounds eluted with hexane (2 ml) and unsaturated compounds containing HBIs collected in a subsequent acetone fraction (3 ml). Sterols were collected of silica columns with Hexane:Methyl Acetate, (4:1 (v/v), 6 ml). Prior to extraction, samples were spiked with an internal standards 9-octylheptadec-8-ene (9–OHD) and 5α-androstan-3β-ol (100 ng each) to permit quantification of HBIs and sterols, respectively.

Analysis of HBIs and sterols was carried out using an Agilent 7890 A GC coupled to a 5975 series mass selective detector and operating conditions^75^. Identification of individual biomarkers was based on their characteristic retention indices and mass spectra, while quantification was achieved by comparison of mass spectral responses of selected ions with those of internal standards, and normalized according to their respected instrumental response factors and the mass of sediment extracted^75^. Prior to analysis, sterol-containing fractions were derivatised with N,O–bis(trimethylsilyl)trifluoroacetamide (BSTFA; 100 ml; 70 °C for 60 min).

### Chronology of Site U1537

Low-resolution age control for Site U1537 is provided by shipboard magnetostratigraphy (brown squares in Fig. 2; ref. ^76^). We used the five magnetic reversals (termination of Olduvai, termination of Cobb Mountain, onset and termination of Jaramillo, and the Matuyama/Brunhes reversal) as first order age control points. In our age modeling, we used the depth range, defined by the last and first occurrence of stable polarity below and above the transition interval. The reversals were identified in both Holes U1537A and U1537D at almost identical depth (Supplementary Table 1 and Supplementary Fig. 1). Successive tuning was not allowed to invalidate the magnetostratigraphic constraints (Supplementary Table 2). Biostratigraphic datums, i.e. the first (FO) and last (LO) occurrence of 18 species (Supplementary Table 1; green diamonds with uncertainties in Fig. 2) provide further stratigraphic ground truth data; however, those were not used as direct tie points due to larger age uncertainties.

The high-resolution age models rely on tuning MS maxima of Site U1537 to dust maxima of the EDC ice core^19^ back to 0.8 Ma, and to heavy δ^18^O values in the LR04 record before that time (see main text). The final, resulting age model for Site U1537 consists of 151 tie points (black and blue dots in Fig. 2; Supplementary Table 2). Note that the measurement interval of 2 cm provides a sample resolution for MS (and most other proxies used here) in the upper decadal to lower centennial band for the high-resolution tuning in step two. As a consequence of the dust (MS) tuning, all maxima in b* (i.e. biogenic opal) are associated with interglacials, i.e. light δ^18^O values in the LR04 stack over the entire record spanning the last 1.5 Ma. Furthermore, the long-term variability in sedimentation rates (SR) shows only one major change around the MBE from ~10 cm/kyr before to ~20 cm/kyr after that time.

We note that tuning other dust proxies such as Ca and Fe^20^ show comparable temporal variability and would produce very similar high-resolution age models (Figs. 3c and 5h, j). However, we chose MS because it reveals the closest resemblance to Antarctic dust (Supplementary Fig. 3).

### Age uncertainty modeling

*Undatable MATLAB* tools were used for age-depth modeling and quantification of age uncertainty, using a “x-factor” of 0.1 (ref. ^77^). Three types of age-control points were used (Extended Data Table 2): tie points between the U1537 MS record and the EDC dust record on the AICC 2012 age scale^26^, tie points between the U1537 MS record and the LR04 stack^23^, and magnetic reversals based on their occurrence at North Atlantic IODP Site U1308 relative to the LR04 timescale^78^. Uncertainties of ±5 kyr were used for the LR04 tie points, together with the variable uncertainties of the AICC 2012 tie points (see Extended Data Table 2), to account for uncertainty in the graphical correlation and phase of the signals. For the magnetic reversals, we use the depth range of the first and last observation of stable polarity as uncertainty, defined as inclinations greater or less than 45°. For the last 1.5 Ma, the U1308 derived ages of magnetic reversals, based on high-resolution benthic δ^18^O and high-resolution paleomagnetic measurements, are better intercalibrated with the LR04 timescale than the 2012 Geomagnetic Polarity Time Scale (GPTS)^79^, particularly for the age of the upper boundary of the Cobb Mountain Subchron, which is 15 kyr younger on the 2012 GPTS than the LR04 timescale^78^.

### Concentration versus accumulation profiles

Concentration profiles for proxies used in this study (MS, Fe, and Ca for dust; b*, opal and Si/Al as well as Si/Ti ratios for paleoproductivity) demonstrate clear glacial to interglacial trends. Do these trends hold if converted, as far as possible, into flux rates? To answer this question one needs to consider that the substantially elevated accumulation rates in the Scotia Sea relative to surrounding areas are achieved through syn-sedimentary focusing of material within Weddell Sea sourced AABW and the overlying ACC. ^230^Th-normalization data for neighboring Site MD07-3134 indicates interglacial focusing factors of 5–13 and glacial focusing factors of 11–29 (ref. ^28^). Due to the radioactive decay, this method is only applicable over ~the last two glacial cycles. Hence, calculation of MAR on our long time series is problematic and will likely lead to erroneous results and misinterpretations.

To explore the relation between concentration and accumulation profiles we calculated MAR by first determining the age depth relationship at MIS boundaries 1–50 (Fig. 5b). To avoid stair-case jumps we then interpolated the resulting SR profiles using a spline function (Fig. 5a). Next, we calculated dry-bulk densities (DBD) by relying on shipboard results obtained on 69 moisture and density discrete samples at Site U1537. We used their average porosities and grain densities and applied an iteration algorithm^72^ to determine DBD for each non-destructive GRA measurement (Fig. 5c). Then we multiplied DBD by the spline SR to calculate MAR (Fig. 5d). For each sediment component we then multiplied the concentration by the MAR.

XRF scanning counts of individual elements have not been calibrated to concentrations through discrete sample XRF measurements for Site U1537. However, such results were obtained for neighboring Site MD07-3134 with a Philips PW1400/1480 XRF spectrometer^20^. At that site, the relationship between scanned XRF counts for Fe and Ca and discrete measurements show a good fit to sample-based concentrations of Fe and Ca, with correlation coefficients of *r* = 0.91 and 0.89, respectively (Supplementary Fig. 2). Fe contents range 3–7%, corresponding to 3000–11,000 counts, and Ca content varies 0.8–2.5%, corresponding to 2000–8000 counts. We used the slope of the linear regressions to calculate Fe and Ca weight %. For the principally higher XRF count range at Site U1537 (80,000–230,000 for Fe; 10,000–50,000 for Ca) we adjusted the slope accordingly, and calculated Fe (%) by multiplying Fe counts by 0.00003 and adding 1.25. For Ca (%) we multiplied Ca counts by 0.00003 and added 0.37. The results were then used to calculate MAR of Ca and Fe (Fig. 5h, j). However, for MS, which has a complex dependence on mineralogy, grain size, and concentration of ferromagnetic minerals, such calibration is not applicable. Therefore, we multiplied the relative concentration given in instrument units by the MAR. The resulting temporal trend is correct, however, the absolute numbers need to be treated with caution.

Although unlikely reflecting the true rain rates of any component—given the strong focusing—the overall results show that all terrigenous proxy MAR calculations for MS, Ca, and Fe (Fig. 5f, h, j) are still very well correlated to ice-core dust and dust proxy fluxes (Fig. 5g, i). This is not surprising because the ^230^Th-normalization data indicates higher glacial focusing, i.e. high glacial concentrations. Also, DBD are generally higher during glacials because fine-grained detrital deposits have lower porosities and higher grain densities than opal-rich, interglacial deposits. Hence, MAR are generally higher during glacials, i.e. the high glacial concentration of terrigenous proxies will lead to even higher glacial MAR of terrigenous proxies. However, substantial highs in opal MAR are mainly preserved during the last four interglacial cycles since the MBE, after which opal contents varied substantially with very high interglacial values (Fig. 6) at times of high interglacial sediment MAR (Fig. 5d). In the earlier record, opal contents varied less and the generally higher glacial MARs further reduce the amplitude in the opal AR record (Fig. 5e).

Overall, we conclude that including MAR calculations does not add vital information, nor does it alter our interpretation. The occurrence of higher terrigenous flux during glacials holds true regardless of comparing concentration or AR profiles to ice cores. In fact, interglacial processes may even be obscured when MAR are included because of generally elevated MAR and more severe sediment focusing during glacials. However, the XRF normalizations which are independent of MAR and focusing considerations confirm interglacially elevated bioproductivity.

### Opal estimation from color reflectance

At neighboring Site MD07-3134, color component b* shows a linear relationship to opal content for the last glacial cycle^28^. This core is 58 m long and located in the same small basin as Site U1537, ca. 40 km to the southwest. At Site U1537, b* values are similarly correlated with a coefficient of *r* = 0.9 (Supplementary Fig. 2a); opal content is elevated during interglacials and lower during glacials with values range from 2–55 wt % (Fig. 6a). We should note that the Ocean Optics QE Pro detector used for IODP cores produced much more noisy data than the Minolta Chromatometer CM-2002 used for MD cores. Therefore, we applied a 15-point running average to smooth the b* data set before calculating opal content (black curve in Fig. 6a).

Accordingly, interglacial periods show high opal contents, up to 55–60 %, especially in the upper part of the U1537 record, whereas glacial periods generally range 0–10 %. The relationship is striking for Site U1537 and holds for the entire record. We emphasize that intervals of elevated estimated opal content are found in all lithostratigraphic descriptions as thick sedimentary packages of diatom oozes with >50% diatoms and <10% spicules as identified in smear slides^16^, especially in the upper part of Site U1537, where each of these interglacial packages is more than 10 m thick, indicative of superproductivity periods. Sections with low estimated opal content (glacials) are described as silty-clay with >25% quartz and <10% opaque minerals.

Gamma-ray density (GRA) has also been used to estimate opal content in neighboring Site MD07-3134 (ref. ^28^). GRA values below ~1.3 g/cm^3^ can only be found in unconsolidated sediments of high porosity and low grain density^80^—conditions usually only met if marine deposits are rich in biogenic opal (Supplementary Fig. 2). According to the lithological description for Site U1537 (ref. ^16^), those sections are exclusively diatom oozes. The temporal variability of GRA at Site U1537 shows such systemically low values only during interglacials, whereas glacial deposits are ≥1.6 g/cm^3^; providing further indication for substantially higher opal contents during interglacials. We should note, however, that we did not attempt to estimate biogenic opal quantitatively from GRA since there is a well-known general down-core trend toward higher values due to increasing compaction and de-watering of the sediment, especially within the upper 120 m (ca. 800 ka; see gray line in Fig. 7d). This is the reason why the inverse relationship between b* and GRA is slightly polynomial, but otherwise similar between Sites MD07-3134 and U1537 (Supplementary Figs. 2 and 3).

Opal dissolution downcore is another potential problem. However, opal contents do not simply decrease downcore as a sign of decreasing preservation/increased dissolution at Site U1537, instead they show plateau-like, long-term changes with relatively high contents in the lowermost section studied here (between MIS 37 and 49). Also, the fact that productivity proxies Si/Al and Si/Ti show the same long-term step changes argues against increasingly poor opal preservation downcore. In addition, the smear slides investigated onboard JR^16^ did not show significant preservation changes in the section presented here. More severely, diagenetic changes could occur deeper in the sediment column, near the opal C/T boundary, which is often in 400–600 m water depth in the circum-Antarctic realm. However, we only report data from the top 200 m (e.g. Supplementary Fig. 1).

The fact that the glacial-to-interglacial pattern is mainly driven by climate-related changes producing opal-rich and terrigenous-rich deposits, is also shown by the variability of NGR. Higher values generally indicate elevated content of radioactive isotopes of potassium (K) thorium (Th) and uranium (U), which are commonly enriched in clay minerals. XRF scanning data also shows that specifically K, Fe, and Ti are elevated for clay-rich sediment and restricted to glacial periods. Hence, clays dominate glacial times at Site U1537 and their high NGR is inversely correlated to GRA. The systematic, high-amplitude glacial-to-interglacial variability is again striking and holds through the entire record (Fig. 7c, d). In summary, all sediment physical property data identify striking lithologic changes with high-amplitude, climate-driven alterations of terrigenous and biogenic deposits on orbital time scales.

## Supplementary information

Supplementary Information

## Data Availability

All raw data of IODP Expedition 382 (Iceberg Alley) are available in the IODP data base LIMS (http://web.iodp.tamu.edu/LORE/) and accessible through the scientific proceedings^16^. All processed data of Site U1537 used in this publication are available in the PANGAEA data base system (10.1594/PANGAEA.939650)^81^.
