# Supplementary material for: Antiphased dust deposition and productivity in the Antarctic Zone over 1.5 million years
Source: Nat Commun. 2022 Apr 19;13:2044. doi: 10.1038/s41467-022-29642-5 (PMC9018689; doi:10.1038/s41467-022-29642-5)
Supplement: Supplementary file 1 — Supplementary Information [file 41467_2022_29642_MOESM1_ESM.pdf]

Supplementary Information for

# Antiphased dust deposition and productivity in the Antarctic Zone over 1.5 million years

Michael E. Weber[1, *], Ian Bailey[2], Sidney R. Hemming[3], Yasmina M. Martos[4, 5], Brendan T. Reilly[6], Thomas A. Ronge[7], Stefanie Brachfeld[8], Trevor Williams[9], Maureen Raymo[3], Simon T. Belt[10], Lukas Smik[10], Hendrik Vogel[11], Victoria Peck[12], Linda Armbrecht[13], Alix Cage[14], Fabricio G. Cardillo[15], Zhiheng Du[16], Gerson Fauth[17], Christopher J. Fogwill[14,18], Marga Garcia[19,20], Marlo Garnsworthy[21], Anna Glüder[22], Michelle Guitard[23], Marcus Gutjahr[24], Iván Hernández-Almeida[25], Frida S. Hoem[26], Ji-Hwan Hwang[27], Mutsumi Iizuka[28], Yuji Kato[29], Bridget Kenlee[30], Suzanne OConnell[31], Lara F. Pérez[32], Osamu Seki[33], Lee Stevens[34], Lisa Tauxe[6], Shubham Tripathi[35], Jonathan Warnock[36], and Xufeng Zheng[37]

## Contents

Supplementary Figures 1–4
Supplementary Tables 1–2
Supplementary References 1–11

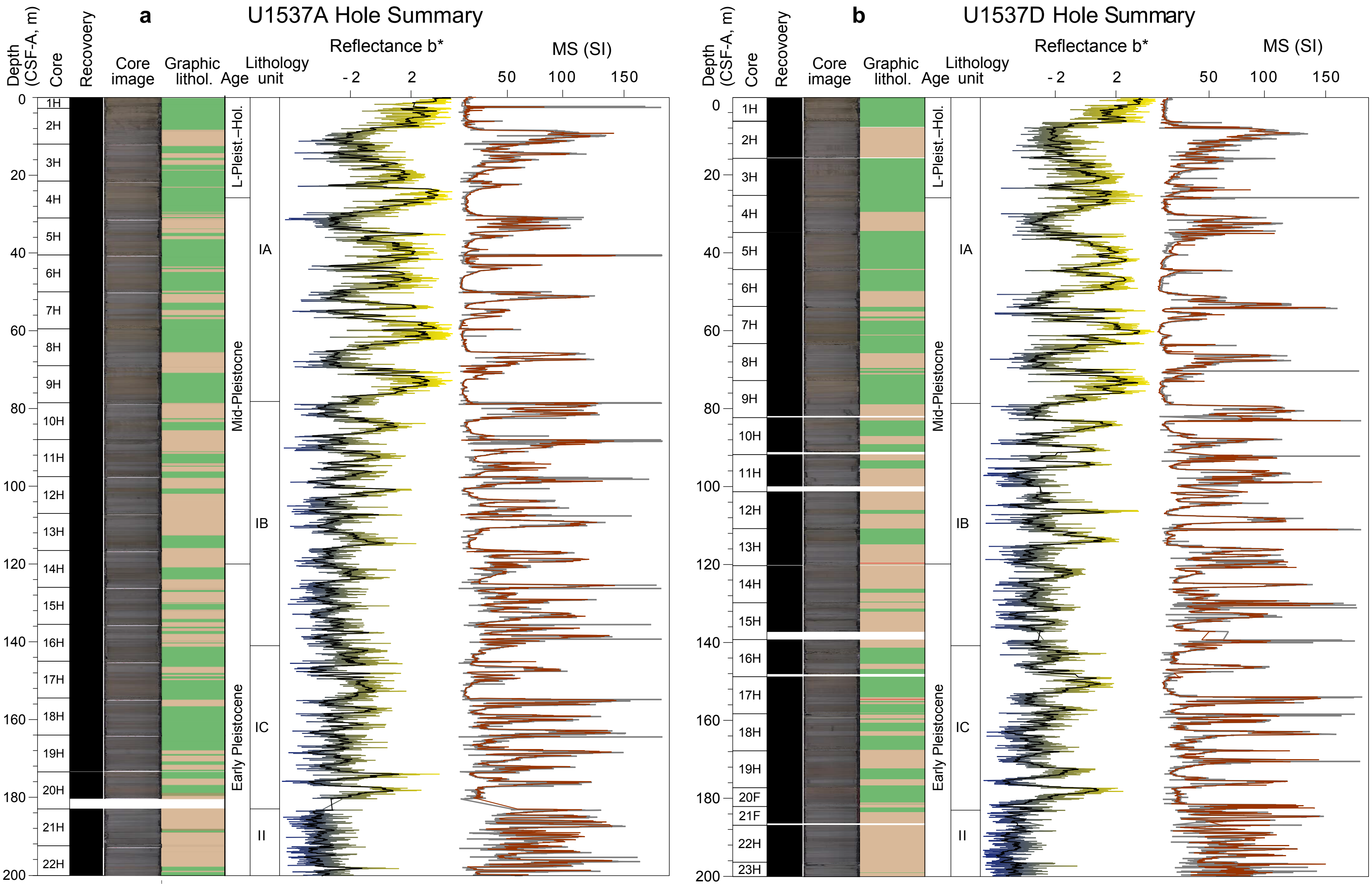


**Supplementary Fig. 1. Hole summary plots for U1537A and U1537D.** Depth, core number, recovery, core image, main lithology (green is diatom ooze; beige is silty clay), age, lithologic unit, color reflectance b* (blue-yellow curve is raw data; black curve is 15-point smoothed average), and magnetic susceptibility (MS; gray is point sensor, red-brown is whole-round measurement) for Holes U1537A (**a**) and U1537D (**b**). Both holes retrieved nearly identical records with very good recovery (close to 100%) at Site U1537 (ref[1]). Note that highs in b* are systematically associated with diatom oozes, whereas highs in MS occur mainly in silty clays.

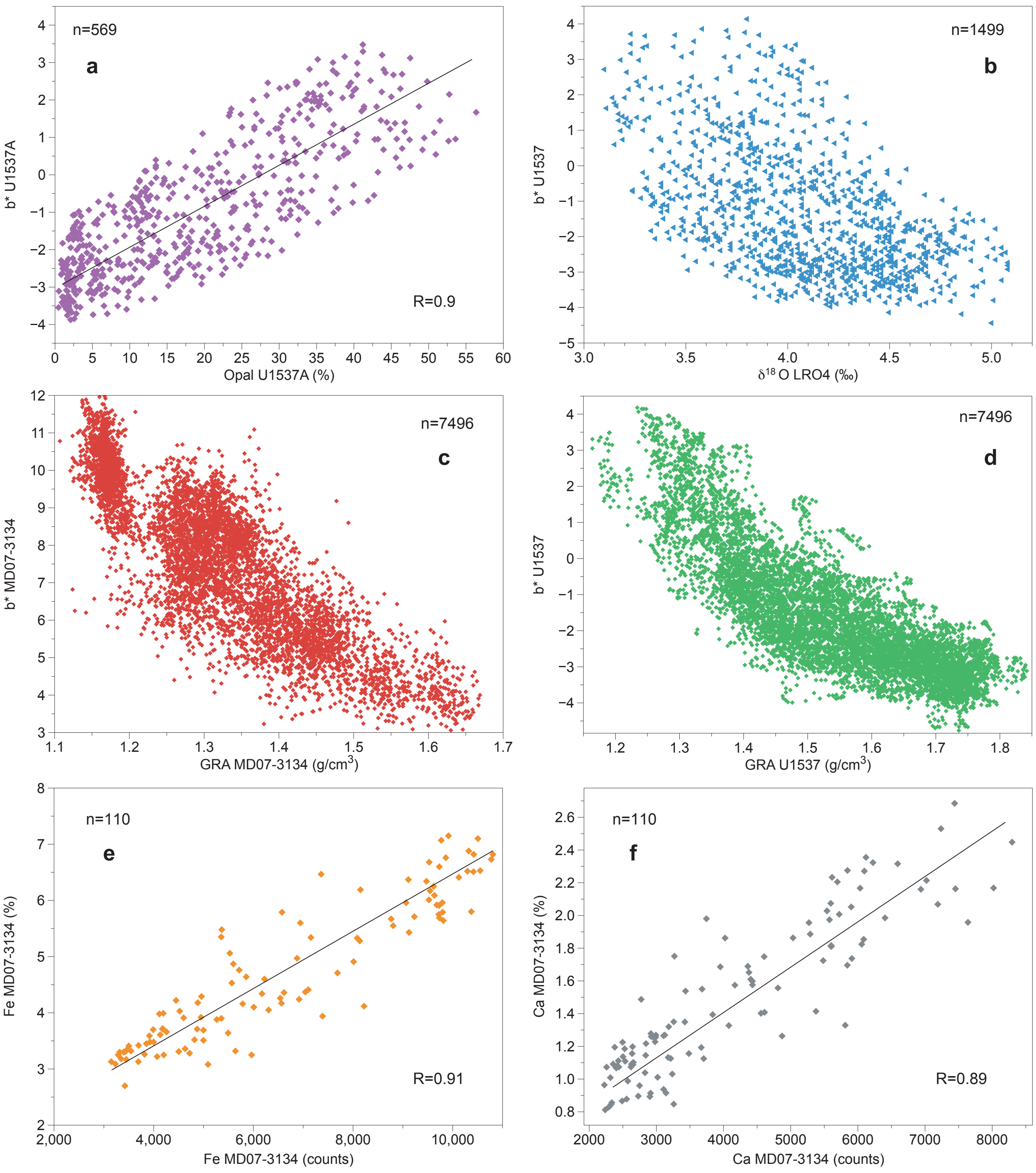


**Supplementary Fig. 2. Cross plots of paleoproductivity and climate proxies. a** Relationship between b* and opal content in Site U1537. Note the good positive linear relationship. **b** Site U1537 b* record and global ice volume stack LR04 (ref[2]). Note the positive but scattered relationship. **c** and **d** show the similarly inverse relationships between b* and gamma-ray density (GRA) for Sites MD07-3134 and U1537, respectively (Site MD3134 is located ~40 km southwest of Site U1537). For Site U1537, values are displayed for the time 0–1.5 Ma. **e, f** Linear relationships between concentration and XRF counts for Fe and Ca, respectively, at Site MD07-3134.

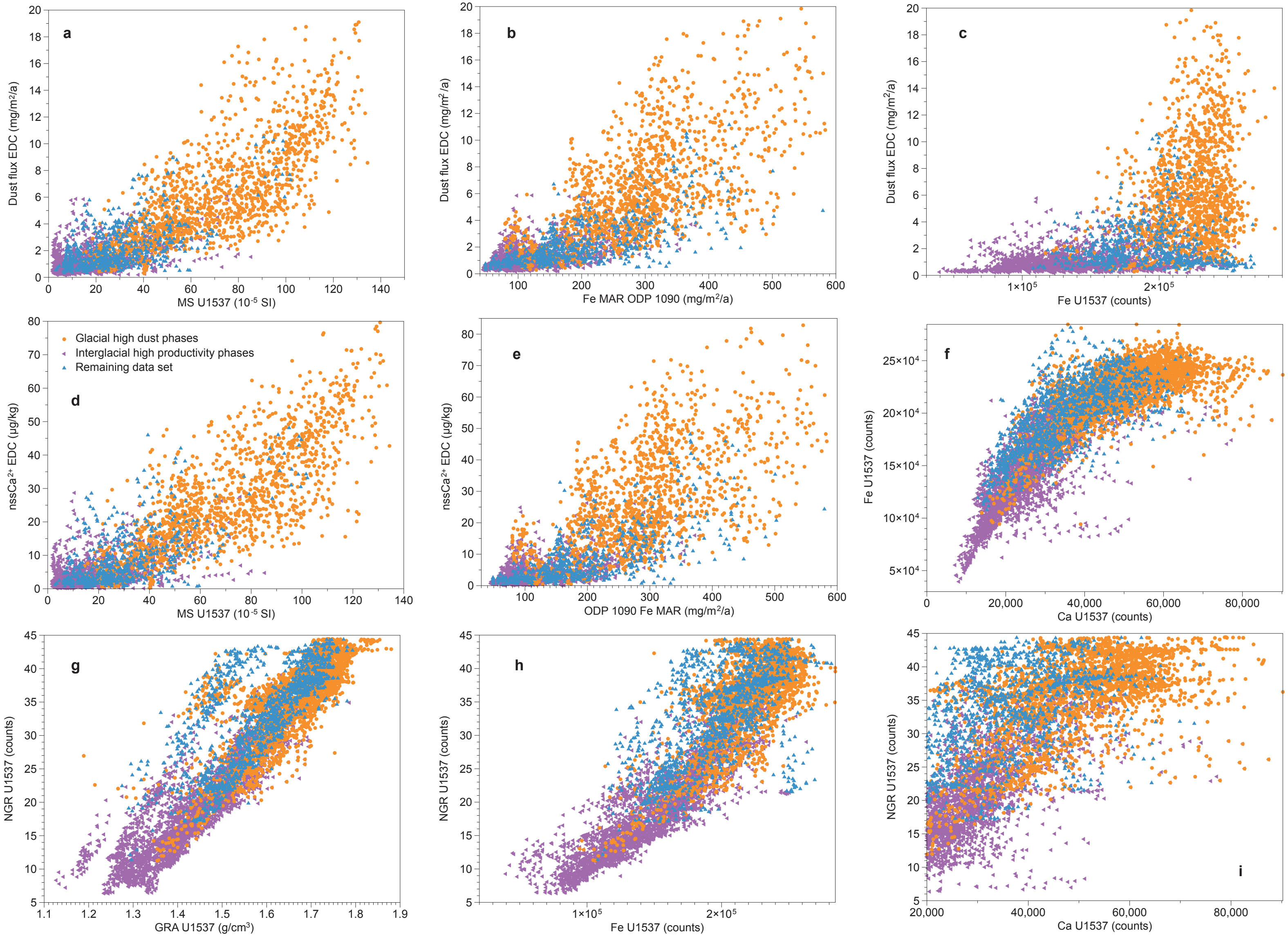


**Supplementary Fig. 3. Cross plots of dust and climate proxies.** Cross plots with data divided into three categories: phases of high glacial dust deposition (orange), phases of enhanced interglacial ocean productivity (purple), and the remaining data set is transitional (blue). **a-c,** Dust flux from the EDC ice core[3] versus U1537 magnetic susceptibility (MS), ODP 1090 Fe mass accumulation rate (MAR), and U1537 Fe counts, respectively. **d-e,** nssCa flux of ice core EDC[4] versus MS of Site U1537 and Fe MAR of ODP Site 1090 (ref[5]), respectively. **f** Fe versus Ca counts of Site U1537. **g-i,** Natural gamma radiation (NGR) of Site U1537 versus gamma-ray density (GRA), Fe and Ca counts, respectively. Note that dust proxy MS of U1537 (**a**, **d**) shows less scattering and a closer, positive relationship to both dust proxies of the EDC ice core (dust flux and nssCa flux) than the Fe MAR of ODP Site 1090 (**b**, **e**). Note further that NGR and GRA of Site U1537 are linearly related (**g**) and depict differences in facies closely. This facies dependence is also partly expressed in the relation of NGR and Fe counts at Site U1537 (**h**) – an additional indication that Fe is not only sensitive to dust but in general to clay-rich, glacial deposits. Values are re-sampled at 0.2 kyr and displayed for the time 0–1.5 Ma (n=7496).

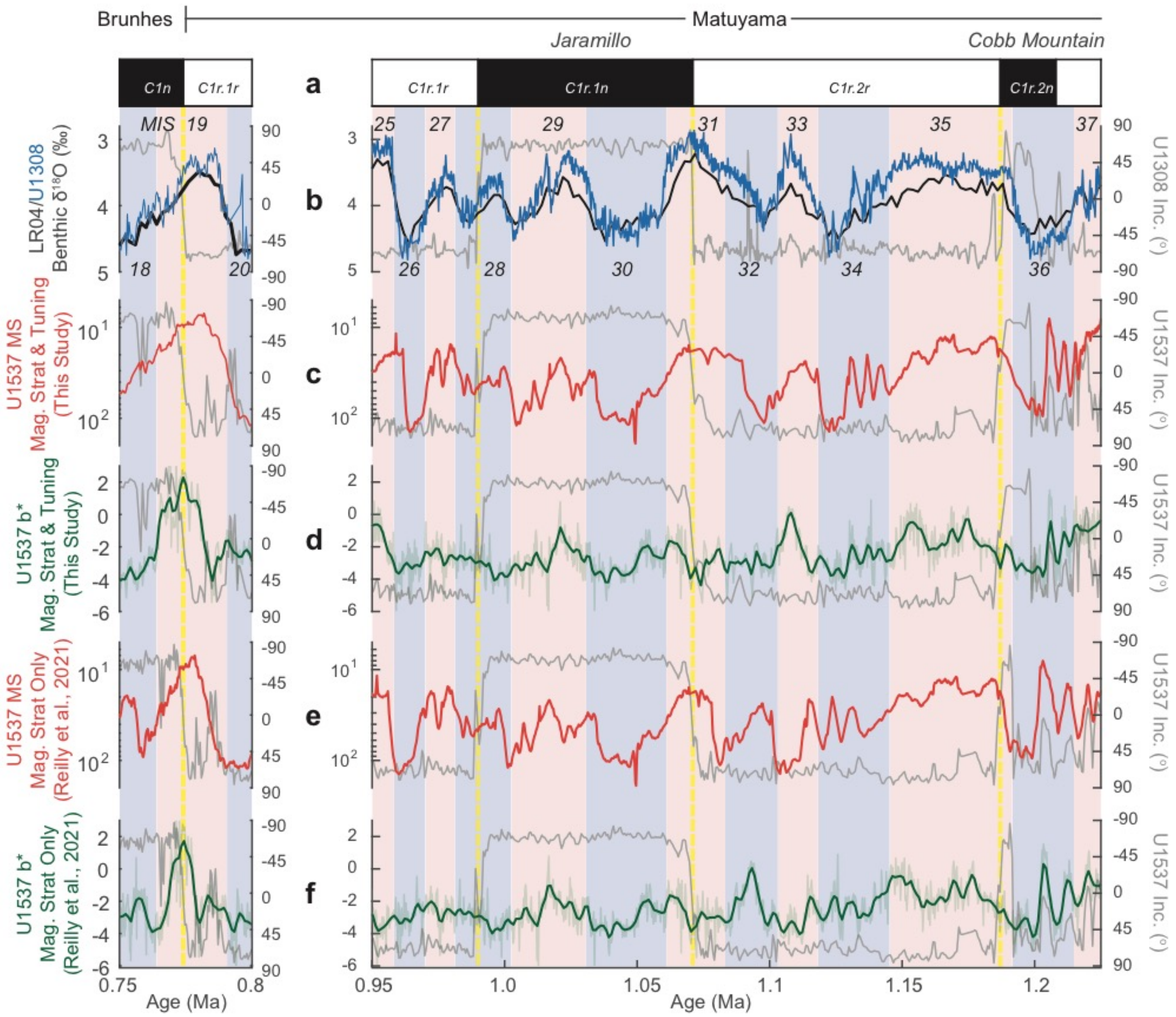


**Supplementary Fig. 4. Tuned and untuned chronologies for the Middle Pleistocene. a** Middle Pleistocene subchrons for the Matuyama and the Matuyama-Brunhes boundary with geomagnetic polarity timescale (black, normal; white, reverse [6]). **b** LR04 benthic $\delta^{18}O$ stack (black; [2]), U1308 benthic $\delta^{18}O$ (blue; [7]), and U1308 inclination (gray; [8]). **c, e** tuned und untuned MS record of U1537, respectively. **d, f** tuned and untuned record of b*, respectively. Red and blue underlain pattern indicates Marine Isotopic Stages (MIS). Magnetic reversals used as age control points are marked by the vertical yellow lines. Details see[9]. Note that the untuned age model indicates that MS highs fall into glacials and b* highs fall into interglacials, providing independent support for the tuning strategy used in this study.

**Supplementary Table 1. Biomagneto-stratigraphy of Site U1537.** Top panels show geomagnetic reversal data and ages[10] for Holes A and D of Site U1537. Note that both holes provide consistent results and identity all reversals at practically identical depths (Supplementary Fig. 1). Lower part shows biostratigraphic datums obtained at Hole U1537A with first (FO) and last (LO) occurrences of diatom (DIAT) and radiolarian (RAD) species (see also[1]).

| | Name | Reversal | Age (Ma) | Core Section Depth (top) | Core Section Depth (bottom) | Top Depth (CSF-A) | Bottom Depth (CSF-A) | Mid Depth (CSF-A) | Top Depth (CCSF, m) | Mid Depth (CCSF, m) | Bottom Depth (CCSF, m) |
|---|---|---|---|---|---|---|---|---|---|---|---|
| *Paleomagnetic data U1537A* | | | | | | | | | | | |
| | Matuyama/Brunhes | C1n (o) | 0.774 | 12H-7, 45 cm | 13H-1, 100 cm | 106.96 | 108.00 | 107.48 | 115.67 | 116.33 | 116.99 |
| | Jaramillo (t) | C1r.1n (y) | 0.990 | 15H-2, 35 cm | 15H-2, 70 cm | 127.87 | 128.22 | 128.05 | 138.86 | 139.04 | 139.21 |
| | Jaramillo (o) | C1r.1n (o) | 1.071 | 15H-7, 55 cm | 16H-1, 85 cm | 135.60 | 136.35 | 135.98 | 146.65 | 147.03 | 147.40 |
| | Cobb Mountain (t) | C1r.2n (y) | 1.187 | 17H-1, 95 cm | 17H-1, 120 cm | 145.95 | 146.20 | 146.08 | 156.24 | 156.36 | 156.49 |
| | Olduvai (t) | C2n (y) | 1.780 | 23H-5, 85 cm | 23H-5, 95 cm | 208.83 | 208.93 | 208.88 | 222.11 | 222.16 | 222.21 |
| *Paleomagnetic data U1537D* | | | | | | | | | | | |
| | Matuyama/Brunhes | C1n (o) | 0.774 | 12H-4, 45 cm | 12H-4, 80 cm | 106.23 | 106.58 | 106.41 | 116.43 | 116.60 | 116.78 |
| | Jaramillo (t) | C1r.1n (y) | 0.990 | 14H-6, 20 cm | 14H-6, 70 cm | 128.00 | 128.50 | 128.25 | 138.87 | 139.12 | 139.37 |
| | Jaramillo (o) | C1r.1n (o) | 1.071 | 15H-4, 135 cm | 15H-5, 20 cm | 135.65 | 136.00 | 135.83 | 146.54 | 146.72 | 146.89 |
| | Cobb Mountain (t) | C1r.2n (y) | 1.187 | 16H-8, 75 cm | 16H-9, 15 cm | 145.22 | 145.59 | 145.41 | 156.27 | 156.45 | 156.64 |
| | Olduvai (t) | C2n (y) | 1.780 | 24H-2, 115 cm | 24H-2, 125 cm | 208.47 | 208.57 | 208.52 | 222.14 | 222.19 | 222.24 |
| *Biostratigraphic data U1537A* | | | | | | | | | | | |
| Type | Event | Taxa | | | | | | | | | |
| DIAT | LO | *Rouxia leventerae* | 0.14 | 4H-3A, 75–76 cm | 4H-4A, 75–76 cm | 25.26 | 26.76 | 26.01 | 26.83 | 27.58 | 28.33 |
| DIAT | LO | *Hemidiscus karstenii* | 0.20 | 6H-1A, 75–76 cm | 6H-CC | 41.26 | 50.26 | 45.76 | 44.44 | 49.60 | 54.77 |
| RAD | LO | *Stylatractus universus* | 0.43 | 7H-CC | 8H-CC | 59.59 | 69.17 | 64.38 | 65.32 | 70.09 | 74.85 |
| DIAT | LO | *Rouxia constricta* | 0.30 | 8H-CC | 9H-CC | 69.17 | 78.96 | 74.06 | 77.88 | 82.91 | 87.94 |
| RAD | LO | *Antarctissa cylindrica* | 0.64 | 9H-CC | 10H-CC | 78.96 | 88.20 | 83.58 | 86.21 | 90.35 | 94.50 |
| DIAT | LO | *Actioncyclus ingens* | 0.60 | 10H-CC | 11H-1A, 50–51 cm | 88.20 | 88.51 | 88.35 | 94.50 | 94.65 | 94.81 |
| DIAT | LO | *Rhizosolenia harwoodii* | 0.60 | 10H-CC | 11H-1A, 50–51 cm | 88.20 | 88.51 | 88.35 | 94.50 | 94.65 | 94.81 |
| DIAT | FO | *Thalassiosira antarctica* | 0.65 | 11H-2A, 50–51 cm | 11H-3A, 50–51 cm | 90.01 | 91.51 | 90.76 | 96.31 | 97.06 | 97.81 |
| DIAT | FO | *Porosira glacialis* | 1.15 | 11H-3A, 50–51 cm | 11H-CC | 91.51 | 94.75 | 93.13 | 100.22 | 101.97 | 103.73 |
| DIAT | LO | *Thalassiosira elliptipora* | 0.70 | 11H-CC | 12H-CC | 97.75 | 107.35 | 102.55 | 106.46 | 111.39 | 116.33 |
| DIAT | LO | *Thalassiosira fasciculata* | 0.90 | 14H-1A, 50–51 cm | 14H-3A, 50–51 cm | 117.01 | 120.01 | 118.51 | 127.47 | 128.97 | 130.47 |
| RAD | LO | *Pterocanium trilobum* | 0.86 | 14H-2W, 120–121 cm | 14H-CC | 120.25 | 126.26 | 123.25 | 130.71 | 133.98 | 137.25 |
| DIAT | FO | *Rouxia constricta* | 1.40 | 15H-CC | 16H-CC | 135.84 | 145.40 | 140.62 | 146.89 | 151.29 | 155.68 |
| DIAT | FO | *Fragilaripsis rhombica* | 1.40 | 16H-CC | 17H-CC | 145.40 | 155.06 | 150.23 | 155.68 | 160.87 | 166.05 |
| DIAT | FO | *Fragilariopsis separanda* | 1.40 | 17H-CC | 18H-CC | 155.06 | 164.35 | 159.70 | 166.81 | 171.35 | 175.89 |
| DIAT | LO | *Fragilariopsis barronii* | 1.30 | 19H-CC | 20H-1A, 50–51 cm | 173.30 | 174.01 | 173.65 | 186.40 | 186.75 | 187.11 |
| DIAT | LO | *Shionodiscus t. v. reimeri* | 1.30 | 20H-2A, 50–51 cm | 20H-3A, 50–51 cm | 175.51 | 177.01 | 176.26 | 188.61 | 189.36 | 190.11 |
| DIAT | LO | *Rouxia antarctica* | 1.50 | 20H-2A, 50–51 cm | 20H-3A, 50–51 cm | 175.51 | 177.01 | 176.26 | 188.61 | 189.36 | 190.11 |

**Supplementary Table 2. High-resolution age model of Site U1537 for the last 1.5 Ma.** Age model relies on 138 tie points generated by tuning magnetic susceptibility (MS) of Site U1537 first to the dust flux record of the EDC ice core for the last 0.8 Ma (0–118.07 m) on the AICC 2012 age scale[11] and then to the LR04 stack (118.28–198.40 m) (ref[2]). Note that depth is given for the splice record (mainly a combination of Holes A and B of Site U1537) in m composite core depth (CCSF).

| Tie point | Depth (CCSF, m) | Age (Ma) | Age error (kyr) | Tie point | Depth (CCSF, m) | Age (Ma) | Age error (kyr) | Tie point | Depth (CCSF, m) | Age (Ma) | Age error (kyr) |
|---|---|---|---|---|---|---|---|---|---|---|---|
| Surface | 0.000 | 0.00000 | 0.00 | EDC Tie | 73.166 | 0.35738 | 3.78 | LR04 Tie | 119.136 | 0.80875 | 5.00 |
| EDC Tie | 5.886 | 0.00845 | 0.26 | EDC Tie | 73.832 | 0.36092 | 3.95 | LR04 Tie | 119.794 | 0.81607 | 5.00 |
| EDC Tie | 8.368 | 0.01799 | 0.71 | EDC Tie | 74.971 | 0.36679 | 4.50 | LR04 Tie | 120.411 | 0.82565 | 5.00 |
| EDC Tie | 9.231 | 0.02326 | 1.02 | EDC Tie | 76.707 | 0.37517 | 4.70 | LR04 Tie | 121.316 | 0.83999 | 5.00 |
| EDC Tie | 9.800 | 0.02609 | 0.93 | EDC Tie | 78.153 | 0.38499 | 3.87 | LR04 Tie | 122.342 | 0.84741 | 5.00 |
| EDC Tie | 10.671 | 0.02921 | 0.78 | EDC Tie | 84.461 | 0.42738 | 3.63 | LR04 Tie | 123.424 | 0.85745 | 5.00 |
| EDC Tie | 10.965 | 0.03066 | 0.89 | EDC Tie | 85.641 | 0.43214 | 4.42 | LR04 Tie | 126.066 | 0.87342 | 5.00 |
| EDC Tie | 11.500 | 0.03492 | 0.86 | EDC Tie | 86.176 | 0.43587 | 4.08 | LR04 Tie | 126.587 | 0.88283 | 5.00 |
| EDC Tie | 11.995 | 0.03718 | 0.74 | EDC Tie | 87.201 | 0.44956 | 3.41 | LR04 Tie | 127.535 | 0.89466 | 5.00 |
| EDC Tie | 12.660 | 0.03974 | 0.66 | EDC Tie | 87.455 | 0.45246 | 3.32 | LR04 Tie | 128.275 | 0.90896 | 5.00 |
| EDC Tie | 13.078 | 0.04239 | 0.75 | EDC Tie | 87.722 | 0.45511 | 3.08 | LR04 Tie | 129.311 | 0.92212 | 5.00 |
| EDC Tie | 13.419 | 0.04413 | 0.74 | EDC Tie | 88.110 | 0.45886 | 3.10 | LR04 Tie | 134.810 | 0.95234 | 5.00 |
| EDC Tie | 13.709 | 0.04733 | 0.80 | EDC Tie | 88.451 | 0.46135 | 3.08 | LR04 Tie | 135.940 | 0.96408 | 5.00 |
| EDC Tie | 13.981 | 0.04856 | 1.03 | EDC Tie | 88.730 | 0.46631 | 2.96 | LR04 Tie | 138.493 | 0.98564 | 5.00 |
| EDC Tie | 14.809 | 0.05416 | 0.88 | EDC Tie | 88.896 | 0.46740 | 2.74 | C1r.1n (y) | 138.871 | 0.99000 | 0.00 |
| EDC Tie | 15.065 | 0.05675 | 0.98 | EDC Tie | 89.827 | 0.47663 | 2.38 | C1r.1n (y) | 139.263 | 0.99000 | 0.00 |
| EDC Tie | 15.449 | 0.05912 | 1.42 | EDC Tie | 94.367 | 0.52399 | 2.59 | LR04 Tie | 140.140 | 1.00456 | 5.00 |
| EDC Tie | 15.849 | 0.06364 | 2.28 | EDC Tie | 95.229 | 0.53226 | 2.94 | LR04 Tie | 141.607 | 1.02217 | 5.00 |
| EDC Tie | 16.383 | 0.06880 | 2.10 | EDC Tie | 95.553 | 0.53665 | 3.25 | LR04 Tie | 143.680 | 1.03771 | 5.00 |
| EDC Tie | 16.675 | 0.07140 | 1.88 | EDC Tie | 95.912 | 0.54195 | 3.11 | C1r.1n (o) | 146.544 | 1.07100 | 0.00 |
| EDC Tie | 17.118 | 0.07358 | 1.94 | EDC Tie | 97.073 | 0.55302 | 2.76 | C1r.1n (o) | 146.894 | 1.07100 | 0.00 |
| EDC Tie | 17.417 | 0.07589 | 1.82 | EDC Tie | 97.420 | 0.55479 | 2.57 | LR04 Tie | 147.546 | 1.09840 | 5.00 |
| EDC Tie | 17.799 | 0.07723 | 1.64 | EDC Tie | 100.195 | 0.58367 | 2.45 | LR04 Tie | 148.581 | 1.10759 | 5.00 |
| EDC Tie | 19.821 | 0.08812 | 1.65 | EDC Tie | 100.589 | 0.58598 | 2.52 | LR04 Tie | 149.459 | 1.12234 | 5.00 |
| EDC Tie | 23.358 | 0.09834 | 1.65 | EDC Tie | 101.596 | 0.59308 | 2.54 | LR04 Tie | 153.433 | 1.16160 | 5.00 |
| EDC Tie | 24.087 | 0.10317 | 1.71 | EDC Tie | 102.173 | 0.59702 | 2.45 | C1r.2n (y) | 156.267 | 1.18700 | 0.00 |
| EDC Tie | 24.742 | 0.10743 | 1.72 | EDC Tie | 104.192 | 0.62594 | 3.08 | C1r.2n (y) | 156.587 | 1.18700 | 0.00 |
| EDC Tie | 30.910 | 0.13235 | 1.83 | EDC Tie | 104.482 | 0.62940 | 4.18 | LR04 Tie | 156.837 | 1.19803 | 5.00 |
| EDC Tie | 32.101 | 0.13769 | 2.54 | EDC Tie | 104.873 | 0.63332 | 4.43 | LR04 Tie | 166.345 | 1.24788 | 5.00 |
| EDC Tie | 32.516 | 0.13863 | 2.78 | EDC Tie | 105.470 | 0.63780 | 4.43 | LR04 Tie | 168.478 | 1.27421 | 5.00 |
| EDC Tie | 33.328 | 0.14649 | 2.94 | EDC Tie | 106.018 | 0.64375 | 4.18 | LR04 Tie | 170.559 | 1.28990 | 5.00 |
| EDC Tie | 33.835 | 0.14889 | 3.43 | EDC Tie | 106.455 | 0.65121 | 4.12 | LR04 Tie | 172.928 | 1.31632 | 5.00 |
| EDC Tie | 33.930 | 0.15173 | 3.76 | EDC Tie | 106.838 | 0.65486 | 3.95 | LR04 Tie | 174.823 | 1.33389 | 5.00 |
| EDC Tie | 34.681 | 0.15998 | 3.63 | EDC Tie | 107.307 | 0.66202 | 3.83 | LR04 Tie | 178.030 | 1.35465 | 5.00 |
| EDC Tie | 34.901 | 0.16264 | 3.39 | EDC Tie | 107.419 | 0.66530 | 3.54 | LR04 Tie | 179.885 | 1.37183 | 5.00 |
| EDC Tie | 35.244 | 0.16564 | 3.10 | EDC Tie | 107.730 | 0.66818 | 3.20 | LR04 Tie | 180.848 | 1.37819 | 5.00 |
| EDC Tie | 35.529 | 0.16810 | 3.02 | EDC Tie | 107.865 | 0.67175 | 2.90 | LR04 Tie | 182.909 | 1.39815 | 5.00 |
| EDC Tie | 35.937 | 0.17268 | 2.99 | EDC Tie | 108.286 | 0.67635 | 2.80 | LR04 Tie | 183.654 | 1.41185 | 5.00 |
| EDC Tie | 36.163 | 0.17701 | 2.66 | EDC Tie | 108.433 | 0.67812 | 2.77 | LR04 Tie | 187.007 | 1.43718 | 5.00 |
| EDC Tie | 36.849 | 0.18241 | 2.59 | EDC Tie | 108.766 | 0.68176 | 2.43 | LR04 Tie | 189.131 | 1.45828 | 5.00 |
| EDC Tie | 38.808 | 0.19287 | 2.45 | EDC Tie | 112.021 | 0.71631 | 2.70 | LR04 Tie | 194.585 | 1.49647 | 5.00 |
| EDC Tie | 39.188 | 0.19524 | 2.02 | EDC Tie | 112.345 | 0.71913 | 3.50 | LR04 Tie | 198.404 | 1.53505 | 5.00 |
| EDC Tie | 42.323 | 0.21586 | 2.28 | EDC Tie | 112.688 | 0.72162 | 3.62 | LR04 Tie | 204.021 | 1.57475 | 5.00 |
| EDC Tie | 46.345 | 0.22471 | 3.02 | EDC Tie | 113.787 | 0.73472 | 3.61 | LR04 Tie | 207.202 | 1.60274 | 5.00 |
| EDC Tie | 47.188 | 0.22974 | 2.63 | EDC Tie | 114.115 | 0.73917 | 3.87 | LR04 Tie | 208.929 | 1.62722 | 5.00 |
| EDC Tie | 52.913 | 0.24814 | 2.31 | EDC Tie | 114.346 | 0.74139 | 3.97 | LR04 Tie | 211.390 | 1.65436 | 5.00 |
| EDC Tie | 53.439 | 0.25245 | 2.89 | EDC Tie | 114.566 | 0.74576 | 3.88 | LR04 Tie | 215.413 | 1.70224 | 5.00 |
| EDC Tie | 54.466 | 0.25976 | 2.80 | EDC Tie | 115.277 | 0.75723 | 3.53 | LR04 Tie | 219.027 | 1.74753 | 5.00 |
| EDC Tie | 55.098 | 0.26583 | 3.05 | C1n (o) | 116.425 | 0.77400 | 0.00 | C2n (y) | 222.063 | 1.78000 | 0.00 |
| EDC Tie | 55.640 | 0.27249 | 3.12 | C1n (o) | 116.775 | 0.77400 | 0.00 | C2n (y) | 222.263 | 1.78000 | 0.00 |
| EDC Tie | 59.372 | 0.29359 | 2.74 | EDC Tie | 117.468 | 0.79149 | 3.80 | | | | |
| EDC Tie | 60.831 | 0.29687 | 2.44 | EDC Tie | 118.070 | 0.79756 | 7.44 | | | | |
| EDC Tie | 71.707 | 0.34457 | 2.71 | LR04 Tie | 118.281 | 0.80002 | 5.00 | | | | |

## Supplementary References


1. Weber ME, Raymo ME, Peck VL, Williams T, Expedition 382 Scientists. Iceberg Alley and Subantarctic Ice and Ocean Dynamics. Proceedings of the International Ocean Discovery Program (2021).

2. Lisiecki LE, Raymo ME. A Pliocene-Pleistocene stack of 57 globally distributed benthic $\delta^{18}O$ records. *Paleoceanography* **20**, PA1003 (2005).

3. Lambert F*, et al.* Dust - climate couplings over the past 800,000 years from the EPICA Dome C ice core. *Nature* **452**, 616-619 (2008).

4. Lambert F, Bigler M, Steffensen JP, Hutterli M, Fischer H. Centennial mineral dust variability in high-resolution ice core data from Dome C, Antarctica. *Clim Past* **8**, 609-623 (2012).

5. Martínez-Garcia A, Rosell-Melé A, Jaccard SL, Geibert W, Sigman DM, Haug GH. Southern Ocean dust-climate coupling over the past four million years. *Nature* **476**, 312-315 (2011).

6. Gradstein FM, Ogg JG, Schmitz MD, Ogg GM. In: *The Geologic Time Scale*). Elsevier (2012).

7. Hodell DA, Channell JET, Curtis JH, Romero OE, Röhl U. Onset of “Hudson Strait” Heinrich events in the eastern North Atlantic at the end of the middle Pleistocene transition (~640 ka)? *Paleoceanography* **23**, (2008).

8. Channell JET, Hodell DA, Curtis JH. Relative paleointensity (RPI) and oxygen isotope stratigraphy at IODP Site U1308: North Atlantic RPI stack for 1.2–2.2 Ma (NARPI-2200) and age of the Olduvai Subchron. *Quaternary Science Reviews* **131**, 1-19 (2016).

9. Reilly BT*, et al.* New Magnetostratigraphic Insights From Iceberg Alley on the Rhythms of Antarctic Climate During the Plio-Pleistocene. *Paleoceanography and Paleoclimatology* **36**, e2020PA003994 (2021).

10. Channell JET, Hodell DA, Curtis JH. Relative paleointensity (RPI) and oxygen isotope stratigraphy at IODP Site U1308: North Atlantic RPI stack for 1.2–2.2 Ma (NARPI-2200) and age of the Olduvai Subchron. *Quaternary Science Reviews* **131, Part A**, 1-19 (2016).

11. Bazin L*, et al.* An optimized multi-proxy, multi-site Antarctic ice and gas orbital chronology (AICC2012): 120–800 ka. *Clim Past* **9**, 1715-1731 (2013).
